# Multisensory task demands temporally extend the causal requirement for visual cortex in perception

**DOI:** 10.1038/s41467-022-30600-4

**Published:** 2022-05-23

**Authors:** Matthijs N. Oude Lohuis, Jean L. Pie, Pietro Marchesi, Jorrit S. Montijn, Christiaan P. J. de Kock, Cyriel M. A. Pennartz, Umberto Olcese

**Affiliations:** 1 Cognitive and Systems Neuroscience, Swammerdam Institute for Life Sciences, University of Amsterdam, Amsterdam, The Netherlands; 2 Research Priority Area Brain and Cognition, University of Amsterdam, Amsterdam, The Netherlands; 3 Department of Integrative Neurophysiology, Center for Neurogenomics and Cognitive Research, VU Amsterdam, Amsterdam, The Netherlands; 4 Cortical Structure and Function, Netherlands Institute for Neuroscience, Amsterdam, The Netherlands

**Keywords:** Sensory processing, Striate cortex, Perception

## Abstract

Primary sensory areas constitute crucial nodes during perceptual decision making. However, it remains unclear to what extent they mainly constitute a feedforward processing step, or rather are continuously involved in a recurrent network together with higher-order areas. We found that the temporal window in which primary visual cortex is required for the detection of identical visual stimuli was extended when task demands were increased via an additional sensory modality that had to be monitored. Late-onset optogenetic inactivation preserved bottom-up, early-onset responses which faithfully encoded stimulus features, and was effective in impairing detection only if it preceded a late, report-related phase of the cortical response. Increasing task demands were marked by longer reaction times and the effect of late optogenetic inactivation scaled with reaction time. Thus, independently of visual stimulus complexity, multisensory task demands determine the temporal requirement for ongoing sensory-related activity in V1, which overlaps with report-related activity.

## Abstract (web summary)

How primary sensory cortices contribute to decision making remains poorly understood. Here the authors report that increasing task demands extend the temporal window in which the primary visual cortex is required for detecting identical stimuli.

## Introduction

During perceptual decision making, stimulus presentation triggers an early response component in primary sensory cortices (driven by thalamic bottom-up input^1^) and, often, a late component, thought to mostly result from recurrent activity through top-down, cross-areal interactions^2–4^. Traditional accounts of how sensory stimuli are transformed into appropriate behavioral outputs have mostly characterized this process in terms of feedforward architectures, where progressively higher-order areas extract sensory features of increasing complexity^5^ to eventually instruct motor output. In the visual cortical system, a fast-acting (<150 ms) feedforward sweep is sufficient for image categorization^6^. Accordingly, deep feedforward neural networks, inspired by this cortical hierarchical architecture, achieve near-human performance in image recognition^7,8^. The function of recurrent architectures has been primarily interpreted in the context of processing ambiguous or complex stimuli, for cognitive processes such as attention, and for consciousness^9–16^. For example, extra-classical receptive field effects in the visual system, such as surround suppression, and separating objects from background, are thought to depend on feedback projections from higher to lower visual areas^17–21^. Perceptual decisions involving figure-ground segregation require recurrent processing^17^, the duration of which becomes longer as a function of visual scene complexity^22^. Recently, a form of late activity in rodent V1 that reflects non-sensory variables such as movement, perceptual report, and arousal^23–28^ was shown to originate in prefrontal areas and progressively involve more posterior areas including sensory cortices^23,27^.

Many hypotheses have been proposed on the function of late, recurrent activity in sensory cortices (including distributed motor command generation and context-dependent sensory processing)^24^, but how it causally contributes to perception is debated. Across primates and rodents, the magnitude of late activity correlates with behavioral reports of perception^3,4,29,30^. Suppressing late activity in the primary somatosensory cortex impairs tactile detection^30^, whereas in primary visual cortex it has been argued that feedforward activity is sufficient for visual discrimination^6,31^. We hypothesize that the cognitive demands of a task, which are captured by the set of task rules as instantiated in an attentional set—see e.g., ref. ^32^—determine the temporal extension of the causal involvement of V1 in perceptual decision making, independently of stimulus complexity. The cognitive load may increase as a consequence of increasing attentional demands, such as when multiple sources of information need to be simultaneously monitored. For instance, integration of visual information with other sensory modalities^12,33,34^ may extend the time required by frontal and pre-motor regions to converge to a decision. This process might reflect an evidence accumulation model^35,36^, i.e., a need to integrate information originating in V1 for longer periods in the case of complex, multisensory tasks. Analogously, the predictive processing framework^19,37,38^ posits that visual and decision-related areas will keep on interacting via recurrent connections to jointly represent sensory stimuli and transform them into appropriate motor responses, performing computations for a time interval that may depend on task demands. Therefore, increasing the cognitive load required to perform a task, for instance by introducing the need to simultaneously monitor multiple sensory modalities, may extend the temporal window during which V1 activity remains causally required for perception, independently of visual stimulus features.

## Results

**Fig. 1:**
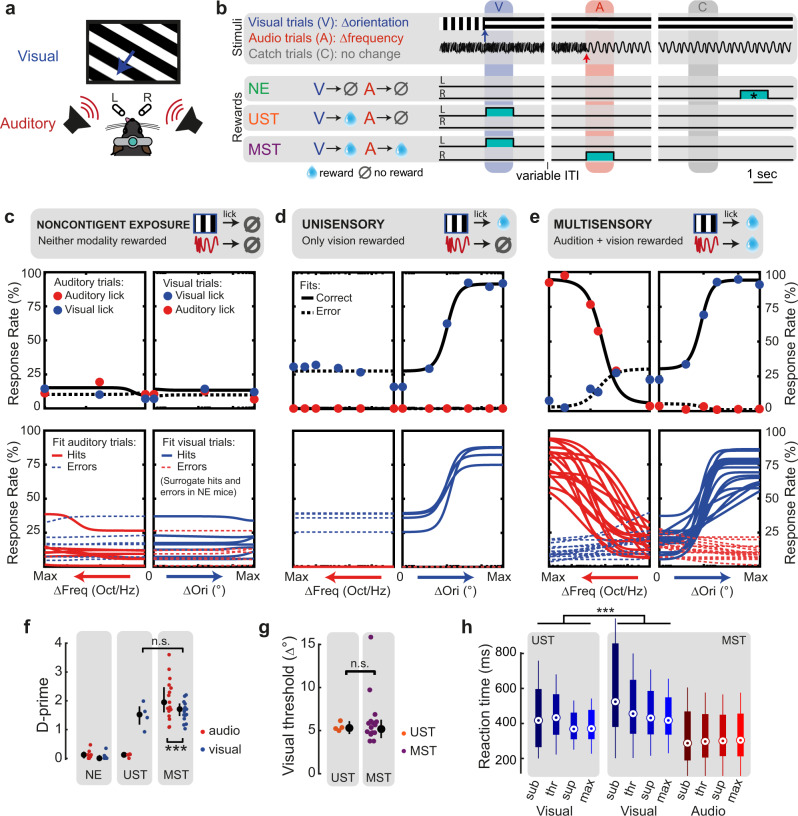
Multisensory task contingencies delay reaction time. **a** Schematic of task setup. **b** Example trial structure with reward availability for each cohort. Three cohorts of mice were presented with the same sensory stimuli: continuous drifting gratings that occasionally changed orientation and direction (visual trial) and a continuous tone that changed frequency content (auditory trial, Supplementary Fig. 1). Cohorts differed in reward structure. Noncontingently exposed (NE) mice were not rewarded contingently to the stimuli. Unisensory trained mice (UST) were rewarded for licks to the left spout after visual trials only, i.e., trained on vision only (cyan blocks denote the reward windows). Multisensory trained mice (MST) were rewarded and trained to lick (for instance) left to report visual changes and right to report auditory changes, i.e., discriminate modality. For NE mice, reward windows were temporally decorrelated from the sensory stimuli, and randomly occurred outside the stimulation period (these windows are denoted as cyan blocks with an asterisk). The trial windows indicate the time window used post-hoc to compare stimulus-related lick rates across cohorts; colors of these windows correspond to the different trial types (blue: visual; red: auditory; gray: catch). For NE mice and auditory trials in UST mice, licks to the visual spout and auditory spout that happened to fall in these windows were defined as surrogate “hits” and “errors” (see Methods). ITI: inter-trial interval. **c** The upper panels show behavioral response rates (dots) and model fits (lines: solid lines for responses to the correct—rewarded—side, dashed lines for responses to wrong—unrewarded—side) for an example session of a noncontingently exposed (NE) mouse. The bottom panels show the average psychometric fits for each mouse obtained by averaging parameters over sessions. Each session was fit with a two-alternative signal detection model (black lines in upper panels, colored in lower panels). **d** Same as **c**, but for UST animals. Note how visual hit rates increase as a function of the amount of visual change, but not auditory change. The relatively high lick rate to the visual spout upon auditory changes arises because only that spout was associated with reward in this task. **e** Same as **c**, but for MST animals. Hit rates increased as a function of both visual and auditory change. **f** D-prime across cohorts. Visual d-prime was comparable for UST and MST (ANOVA, *n* = 151 sessions, *F*(1,29) = 1.60, *p* = 0.22,), and lower than auditory d-prime (ANOVA, *n* = 139, *F*(1,261) = 36.26, *p* = 5.84 × 10^−9^). Each dot is the average over sessions for each animal. Error bars denote the median and interquartile ranges. **g** The detection threshold for visual orientation changes was comparable for UST and MST (ANOVA, *n* = 151 sessions, *F*(1,31) = 0.45, *p* = 0.51). **h** Reaction time for the same subjectively salient visual stimuli (see Methods) was significantly shorter for UST compared to MST (ANOVA, *n* = 3917 trials, *F*(1,3865) = 60.1, *p* = 1.11 × 10^−14^). Saliency levels: sub = subthreshold, thr = threshold, sup = suprathreshold, max = maximal change. Boxplot: dot, median; box limits, 25th and 75th quartiles; whiskers, 1 × interquartile range. **p < 0.01, ***p < 0.001.

To address this hypothesis, we trained mice in three versions of an audiovisual change detection task (task A) with the same stimulus configurations, but different reward contingencies. Head-fixed mice were presented with a continuous audiovisual stream of inputs with occasional instantaneous changes in the orientation of the drifting grating (visual trial) or the frequency of harmonic tones in a ‘Shepard’ stimulus^39^ (auditory trial, Fig. 1a, b; see Supplementary Fig. 1 for auditory stimulus details). We varied the amount of orientation change (visual saliency) and frequency change (auditory saliency) across each animal’s perceptual threshold and fit all behavioral data according to a psychometric multi-alternative signal detection framework^40^. We implemented three distinct task contingencies. First, for noncontingently exposed mice (NE, *n* = 7) neither vision nor audition was predictive of reward, and these mice did not selectively respond to the stimuli (Fig. 1c). In a second version, only vision was associated with reward, and these unisensory-trained mice (UST, *n* = 4) were thus trained to selectively respond to visual changes only, and ignore auditory changes (Fig. 1d). Third, multisensory-trained mice (MST, *n* = 17) were trained to detect both visual and auditory changes (Fig. 1e; e.g., lick left for vision, lick right for audition). Phrased differently, all mice were presented with the same stimuli during training and testing, but lick responses to visual changes were only rewarded in UST and MST mice, and auditory changes only in MST mice. Trials with a stimulus change are indicated as “hit” when the mouse licked the correct spout and “miss” when no lick was provided; error trials correspond to trials with a lick towards the incorrect spout. To compare across cohorts, we also defined (surrogate) hit and miss trials for NE mice, based on whether (unrewarded) licks were performed after stimulus change. In all cohorts, mice performed many trials (mean 569, range 210–1047 per session). The discriminability index (d-prime) was high only for rewarded contingencies, in both the auditory and visual modality (Fig. 1f; for individual mice, see Supplementary Fig. 2).

### Multisensory task contingencies delay reaction time

First, we wondered if visual performance was similar in the unisensory and multisensory task variants (UST and MST) and whether the more complex task contingency slowed responses. There were no significant differences between the cohorts for either maximum d-prime (Fig. 1f), discrimination threshold (Fig. 1g), or sensitivity (for statistics, see Supplementary Table 1). Reaction time, however, did vary across conditions (Fig. 1h). MST mice showed shorter auditory than visual reaction times and reaction times decreased with increasing levels of stimulus saliency for both UST and MST. For the same visual stimuli, reaction time was significantly longer for MST than for UST mice. For both vision and audition, reaction time negatively correlated with performance (Supplementary Fig. 2f, g). The addition of auditory task relevance thus increases reaction times for the same visual stimuli. This result was expected because MST mice were trained to make binary decisions on whether auditory versus visual changes took place, which requires comparisons across sensory channels^41^.

### Early and late activity emerges in V1 of trained mice

**Fig. 2:**
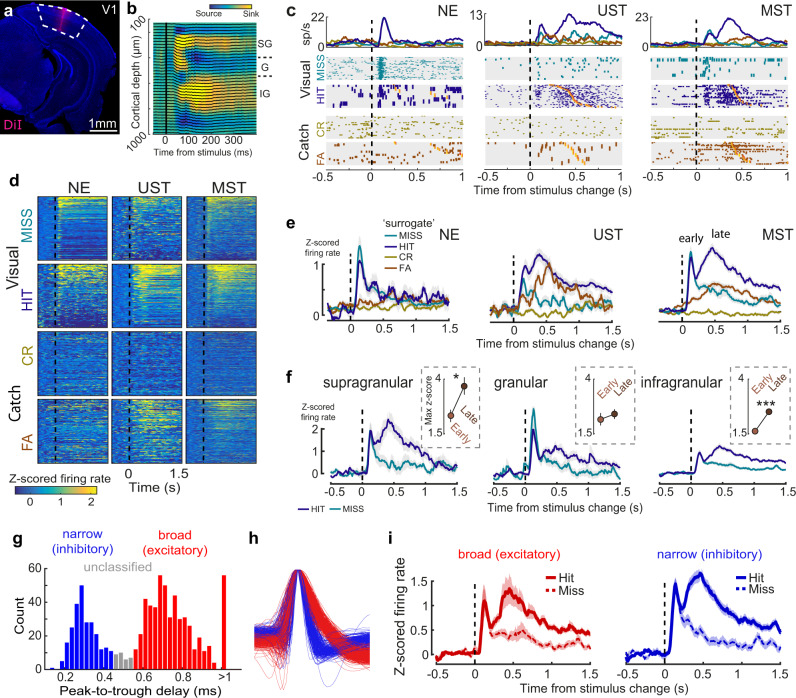
Multisensory task demands modulate late activity in V1. **a** Coronal histological section (3.6 mm posterior to bregma) showing silicon probe tract after recording in V1. **b** Representative example of current source density (CSD) map and LFP traces for checkerboard stimulation. SG = supragranular, G = granular, IG = infragranular. (CSD analysis was repeated with similar results for all 28 mice to determine the depth of probe insertion). **c** Raster plots (bottom) and firing rate (top panel) show stimulus-evoked and report-related activity in three example neurons recorded in V1 (Task A). Raster plots are grouped by trial type (visual or catch) and choice. Within trial type, trials are sorted by post-change orientation and response latency (orange ticks). Note that hits and misses in NE mice are surrogate conditions and are defined post-hoc. CR = correct rejection. FA = visual false alarm. **d** Heatmaps of trial-averaged z-scored activity of all neurons for the three cohorts for the same conditions as in **b**. NE neurons: *n* = 159; UST neurons: *n* = 128; MST neurons: *n* = 510. **e** Averaging z-scored firing rate overall neurons for visual and catch trials split by choice reveals biphasic activity in visual hits but not misses, with late activity only present in animals for which visual trials were rewarded (UST and MST). Note the increase in firing rates in FA trials for UST and MST mice but not NE mice. The weak early transient activity during FA trials in UST and MST mice during this noisy change detection task might be the result of stochastic variability interpreted as a sensory signal, i.e., falsely perceived changes^96^, although a motor (lick) related signal cannot be excluded. See Supplementary Fig. 3 for an in-depth analysis of the lick-related nature of these responses. **f** Same as **e**, but for each laminar zone (neurons from UST and MST mice combined). Inset: maximum z-score during the early (0–200 ms) and late (200–1000 ms) phase of visual hits (SG: *F*(1,194) = 4.60, *p* = 0.03; G: *F*(1,171) = 0.00, *p* = 1.00; IG: *F*(1,1284) = 23.32, *p* < 0.001, ANOVA). **g** Histogram of peak-to-trough delay for all neurons (n = 816 neurons) colored by cell type class: narrow-spiking (peak-to-trough delay <0.45 ms; putative inhibitory; blue) and broad-spiking (peak-to-trough delay >0.55 ms; putative excitatory; red). Single units with intermediate peak-to-trough values were unclassified. The peak-to-trough delay was capped at 1 ms for neurons whose trough extended beyond the sampled window. **h** Normalized average waveform for all V1 neurons colored by cell type class. **i** Z-scored activity averaged over all broad-spiking V1 neurons (left, *n* = 421 neurons) or narrow-spiking neurons (right, *n* = 202 neurons) across UST and MST mice, split by hit/miss response for maximum visual change trials only. Throughout the figure, lines and shading are mean ± SEM.

To investigate whether delayed reaction times corresponded with slower dynamics of late V1 activity, we performed laminar recordings and sampled single-unit activity across cohorts (Fig. 2a). We used the current source density profile in response to visual stimulation and multi-unit activity profile to estimate recorded depth along with the cortical layers (Fig. 2b). In NE animals the instantaneous orientation change evoked a short transient activity in V1 (until about 200 ms after stimulus onset) with a short-lasting tail (Fig. 2c). In visually trained animals (UST and MST), a similar transient wave occurred, but now also a late, second wave of activity was present (emerging around 200 ms after stimulus onset), primarily in hits and to a lesser extent in false alarms (Fig. 2d, e). These dynamics of early and late wave activity were seen for both threshold-level (thr) and maximal (max) orientation changes (Supplementary Fig. 3a, b). Splitting neurons based on recorded depth revealed different laminar dynamics. Early sensory-induced activity was most prominent in the granular and supragranular layers and was similar for hits and misses (*p* > 0.05 for all laminar zones). During visual hits, late activity was prominent in supragranular and infragranular layers and was stronger than early activity (Fig. 2f). The late hit-related modulation (hits–misses, subtracted z-scored firing rate during 200–1000 ms) was stronger in supra- and infragranular layers than in the granular layer (F(2,771) = 4, *p* = 0.019, ANOVA; Posthoc comparison: IG vs G: *F*(1,784) = 12.97, *p* < 0.001; G vs SG: *F*(1,784) = 6.50, *p* = 0.01; IG vs SG: *F*(1,784) = 0.58, *p* = 0.45. This is consistent with the idea that the granular layer is more strongly driven by thalamocortical afferents and extragranular layers more by recurrent processing. We also classified single units based on the delay between peak voltage deflection and subsequent trough. The histogram of peak-to-trough delay showed a bimodal distribution allowing clear classification into narrow and broad-spiking cell types (Fig. 2g, h). The dynamics of early and late components were present in both cell classes (Fig. 2i), suggesting a balanced increase in both excitatory and inhibitory activity upon hits.

### Neural coding during late V1 activity

**Fig. 3:**
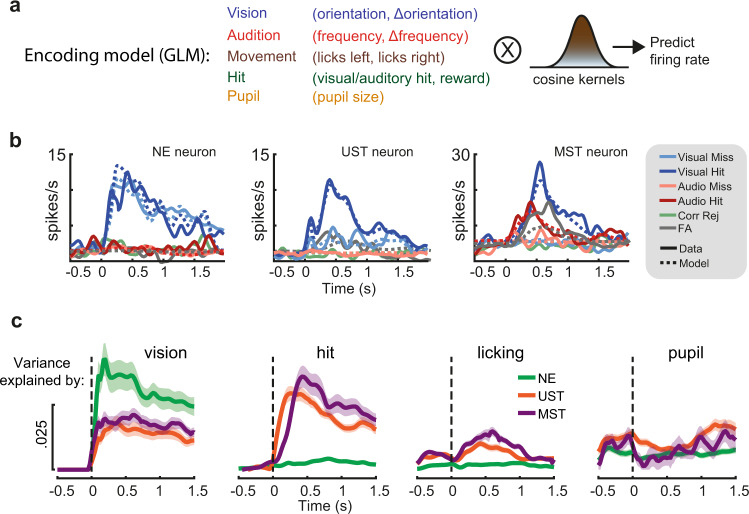
A generalized linear model dissociates time-varying encoding during late activity. **a** We constructed a kernel-based GLM encoding model in which variables related to the sensory environment, task, and behavioral state were included as predictors of firing rate. Binary task variables were convolved with raised cosine basis functions that spanned the relevant time window to model transient firing rate dynamics. **b** Model fits for three example neurons (one V1 neuron from each task version) show that predicted and actual firing rates closely overlap and that both sensory-driven activity (example #1), as well as report-related activity for both visual and auditory hits (examples #2–3), are captured by the model. The five stimulus-response combinations that had the most counts are plotted (trials with false alarms and licks to the incorrect spout are omitted). **c** Explained variance over time for subsets of predictors. Each line shows how much firing rate variance is explained for each time bin across trials based on only including a subset of all predictors. Shaded area corresponds to s.e.m.

Recent studies have shown that late activity in V1 can reflect movement-related variables^25,26,28^. We aligned population activity to the first lick after stimulus onset and found that spiking activity across many neurons was indeed modulated by licking movements, specifically in UST and MST mice (Supplementary Fig. 3c–j). The amplitude of this modulation was higher in trials with correct versus incorrect licks. To further disentangle the contribution of stimulus variables (visual and auditory features and amount of change), movement-related variables (the timing and number of lick responses), hits (visual and auditory hits), and arousal (pupil size), we built a kernel-based generalized linear model (GLM)^27,42,43^ where we included these variables as predictors of firing rate (Fig. 3a; see Supplementary Fig. 4a, b for model performance). The encoding model predicted firing rate dynamics of V1 neurons over time (Fig. 3b) and to investigate the contribution of each of the variables we computed the cross-validated variance of firing rate explained over time by each of these subsets of predictors (Fig. 3c). In NE mice, visual predictors explained most of the variance with negligible contributions from other variables. In UST and MST mice, besides visual predictors, we found that both licking movement and hits explained a significant fraction of variance (Fig. 3c, see also Supplementary Fig. 4c, d). In sum, late V1 activity reflected a combination of visual-, movement- (licking), and hit-related variables, but only in trained mice.

### Multisensory context delays the time course of late activity

**Fig. 4:**
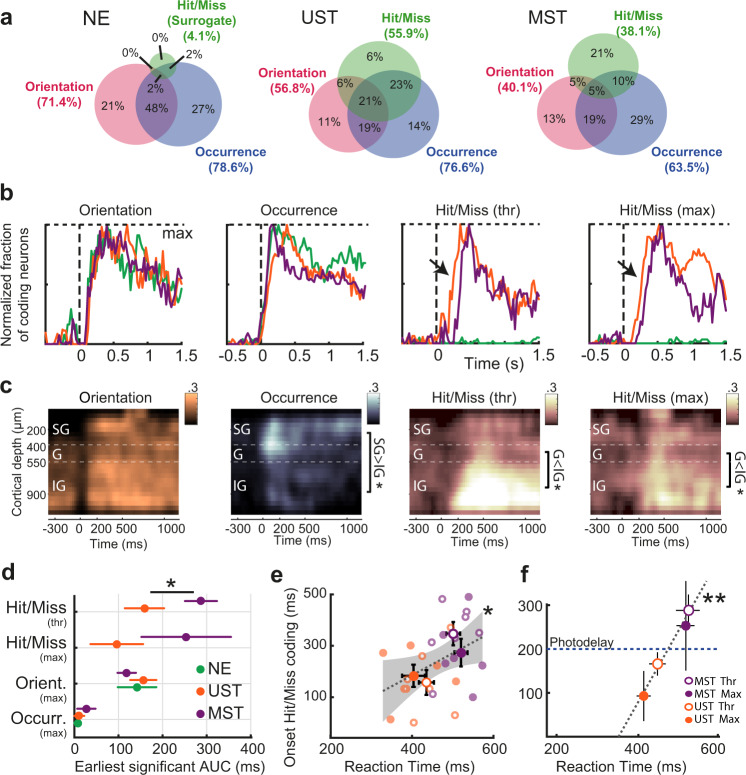
The onset of late activity is delayed in MST mice. **a** The Venn diagrams show for each training cohort the percentage of neurons encoding orientation (grating after stimulus change), occurrence (presence of a visual change or not), or hit/miss (visual hits versus visual misses, with no lick response) as established with ROC analysis. Only maximum visual change trials were used. Shown are percentages out of all coding neurons; percentage of non-coding neurons per cohort: NE: 15.5%; UST: 13.3%, MST: 35.6%. **b** Fraction of neurons (summed over all recordings) coding for task-relevant variables over time. Each coding fraction is baseline-subtracted and normalized by its maximum. Visual hit/miss coding (hits vs misses) was only present in UST and MST mice (as expected) and started earlier in UST than MST mice (highlighted with black arrows). **c** Heatmaps of the fraction of coding neurons across time and cortical depth, with neurons binned based on their recorded depth relative to the granular layer (400–550 μm from dura). Only UST and MST cohorts were included to compare sensory and hit/miss coding in the same datasets. SG = supragranular, G = granular, IG = infragranular. Occurrence coding, 0–200 ms, ANOVA, SG versus IG, *F*(1,16) = 7.21, *p* = 0.016. Hit/miss coding, Thr, 200–1000 ms, G vs IG, *F*(1,15) = 5.21, *p* = 0.037; Max, G vs IG, *F*(1,15) = 4.96, *p* = 0.042. Significance (sidebars): *p < 0.05. **d** Earliest increase in the fraction of significantly coding neurons. Mean ± 95% CI (bootstrap). The apparent fast onset of visual occurrence coding is likely due to temporal smoothing of firing rates. (Bootstrap test, two-sided, UST *n* = 128, MST *n* = 306 neurons, *p* = 0.012) **e** Reaction time correlated with the onset of hit/miss coding in population-averaged activity (ANOVA, *n* = 26 sessions, *F*(1,24) = 5.15, *p* = 0.03). Gray dotted line shows linear regression fit. Each dot is one session. Error bars show mean ± SEM. **f** Same as **e**, but now for the bootstrapped average for each visually trained condition using single-neuron AUC (as in **d**). Reaction time correlated with the earliest moment of significant hit/miss coding (ANOVA, *F*(1,2) = 102.33, *p* = 0.0096). Error bars show bootstrapped mean and 95% CI. Blue dotted line at 200 ms marks the time point where late photostimulation was applied (see Fig. 5d). At this point, unisensory trained mice already showed hit/miss- coding in V1, while multisensory trained mice did not.

To quantify in more detail how the delayed reaction time in MST mice was associated with temporal coding dynamics of single neurons we used a receiver operating characteristic (ROC) analysis^44,45^. Across task versions, the ratio of neurons coding for visual features (grating orientation, and occurrence of a visual stimulus change—i.e., visual trials versus trials with no stimulus change, catch trials) was similar across cohorts (Fig. 4a). In UST and MST mice, however, visual report (i.e., visual hits vs. visual misses, henceforth hit/miss) was also encoded by a substantial fraction of neurons, in line with the averaged z-scored activity (Fig. 2e) and our regression model (Fig. 3). To understand at which time points visual features and hit/miss coding could be read out, we plotted the fraction of neurons that significantly coded for each of these variables over time (Fig. 4b, Supplementary Fig. 5). Temporal dynamics were strikingly similar across cohorts for sensory variables, while hit/miss coding appeared later in V1 for MST than UST mice. When we binned neurons based on their recorded cortical depth, we found that orientation coding was present across cortical layers and the coding of visual change occurrence was confined to an early transient wave in granular and supragranular layers (Fig. 4c). In contrast, hit/miss coding during late activity was predominant in infragranular layers. This spatial segregation with coding of visual change predominant in superficial layers and hit/miss coding in deeper layers suggests that these two processes have different neural substrates (see also Fig. 2f). We quantified the earliest moment of a significant increase in the fraction of coding neurons relative to baseline and found that only hit/miss coding was delayed in MST compared to UST (Fig. 4d; threshold changes: 288 ms ± 36 ms versus 162 ± 28 ms, MST vs. UST, *p* < 0.05; maximal changes: 249 ± 104 ms vs 92 ± 56 ms, MST vs. UST, n.s.). In relation to the delayed visual reaction times in MST mice, we found that the onset of hit/miss coding correlated with reaction time at the level of population-averaged firing rate differences per session (Fig. 4e), as well as the bootstrapped estimate from single neurons across sessions (Fig. 4f). This result was also confirmed by a GLM-based analysis (Supplementary Fig. 4e). Hit/miss-related activity preceded the first lick by about 280 ms (Fig. 4f, Methods). Therefore, at 200 ms after stimulus onset (blue dotted line in Fig. 4f) UST mice generally showed hit/miss coding in V1, while MST mice did not.

### Late activity is causally required for perceptual decision making

**Fig. 5:**
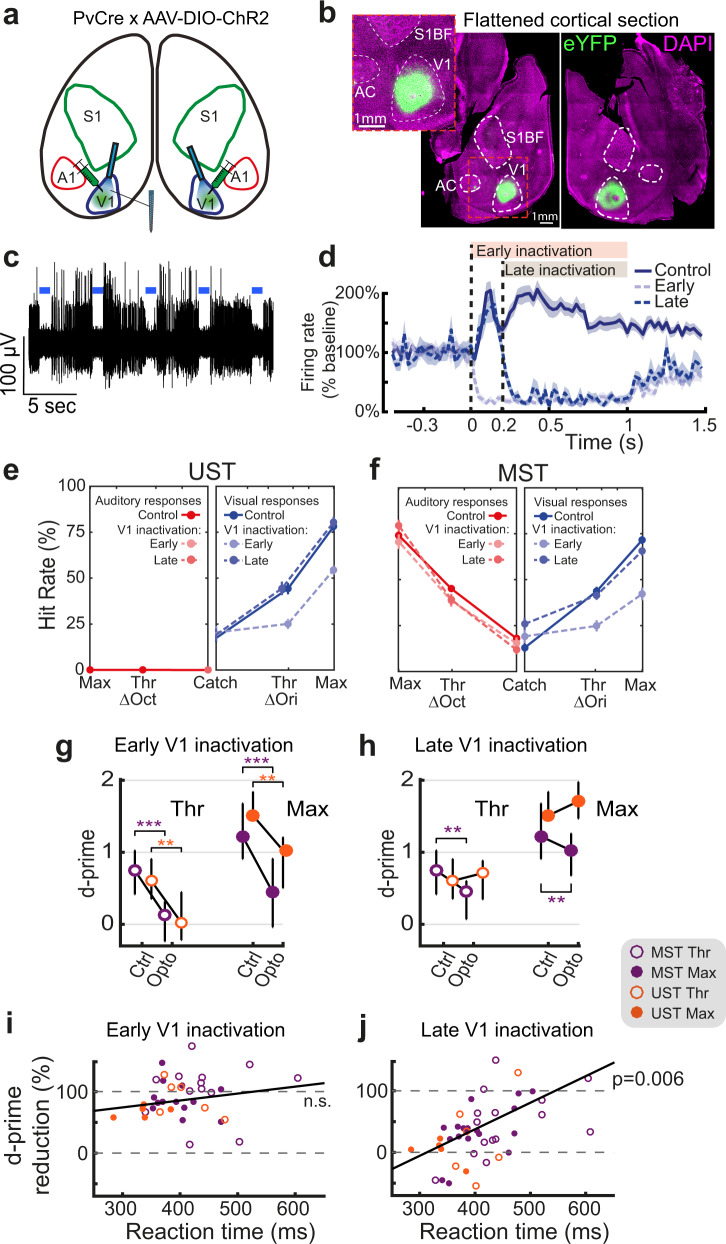
Late silencing of V1 selectively impairs task performance of sessions with slow reaction time. **a** Cre-dependent ChR2 expression in bilateral V1 of PvCre mice allowed robust silencing by locally enhancing PV-mediated inhibition. A1 = auditory cortex, S1 = primary somatosensory cortex. **b** Dorsal view of flattened cortical hemispheres sectioned approximately through layer 4 showing localized viral expression in bilateral V1. (Repeated with similar results for all 28 mice) **c** High-pass filtered trace from an example V1 recording site showing robust silencing of multi-unit spiking activity during bouts of 1-s photostimulation (blue bars). **d** Baseline-normalized firing rate averaged over V1 neurons from UST and MST mice. Control trials are visual hits. Mean ± SEM. **e** Behavioral response rates for control, early, and late silencing trials follow plotting conventions of Fig. 1c–e. **f** Same as **e**, but for MST mice. Both early and late silencing affected visual change detection rates. For the increase in FA see Methods. **g** Early silencing affected visual discrimination performance (d-prime) for both saliencies across UST and MST cohorts. (ANOVA, UST *n* = 18, MST *n* = 34 sessions, UST Thr, *F*(1,32) = 16.71, *p* = 0.0032; UST Max, *F*(1,32) = 14.80, *p* = 0.0064; MST Thr, *F*(1,59) = 35.32, *p* = 2 × 10^−6^; MST Max, *F*(1,58) = 32.56, *p* = 5 × 10^−6^, each corrected for four multiple comparisons (Bonferroni-Holm)) **h** Effect of late silencing depended on task type: late silencing only reduced d-prime in MST (same *n* as **g**, ANOVA, Thr, *F*(1,54) = 13.90, *p* = 0.00553, Max, *F*(1,53) = 13.48, *p* = 0.0067), but not UST mice (Thr, *F*(1,32) = 0.29, *p* = 1, Max, *F*(1,30) = 1.19, *p* = 0.85). For both **g** and **h**, *p < 0.05, **p < 0.01,***p < 0.001, errorbars denote inter-quartile range. **i** The effect of early silencing (quantified as the reduction in d-prime) was not significantly correlated with the median reaction time in control trials from the same session (ANOVA, *n* = 40 conditions, *F*(1,33) = 1.71, *r* = 0.048, *p* = 0.865). **j** Same as **i** but for late silencing. The effect of late silencing was significantly correlated with the reaction time (*n* = 45, F(1,15) = 10.04, *r* = 0.423, *p* = 0.03).

Next, we wondered whether V1 activity occurring after the onset of report-related activity could be causally linked to perception. We locally expressed ChR2 in parvalbumin-expressing interneurons in V1 (Fig. 5a, b, Supplementary Fig. 6a) to achieve temporally specific optogenetic inactivation^46,47^. Laser stimulation robustly silenced multiunit activity (Fig. 5c). To determine the temporal window of V1 involvement, we silenced it either from the moment of stimulus change onwards (“Early”, 0 ms) or from the 200 ms temporal cutoff we identified in the onset of hit/miss coding in UST and MST mice (“Late”, 200 ms; Fig. 4f). Photostimulation was performed during a subset of all trial types (including catch trials, probing the effect of photostimulation without any relationship to stimuli or motor responses) and continued until the animal made a choice. Early blue light stimulation during visual trials (i.e., starting at the onset of stimulus change) reduced the activity of putative excitatory neurons to about 5% of their baseline activity. Late photostimulation left the initial sensory-driven response intact but silenced activity after 200 ms relative to stimulus onset (Fig. 5d).

Early silencing of V1 strongly reduced detection of orientation changes during both UST and MST task performance (Fig. 5e, g), consistent with the primary role of V1 in visual feature processing^31,48,49^. For threshold levels of orientation change (Fig. 5g), the detection of visual change was fully suppressed, indicating that early inactivation of V1 is sufficient to impair visual change detection. Interestingly, late silencing only affected the change detection performance of MST mice (Fig. 5f, h). V1 silencing did not affect auditory change detection (Supplementary Fig. 6b). Moreover, photoillumination of control area S1 did not affect visual or auditory performance (Supplementary Fig. 7).

Even though late silencing impaired visual change detection in MST mice *on average*, results across animals and experimental sessions were mixed: some sessions showed robust behavioral impairment, whereas others showed little effect (Supplementary Fig. 6c, f). We hypothesized that this variability could relate to changes in the speed of the perceptual decision-making process, with sessions having slow reaction times being proportionally more affected due to extended reliance on V1. In fact, this could be observed when we separately analyzed the behavioral consequences of optogenetic inactivation on the fastest and slowest sessions (top and bottom 50% of all sessions split by mean reaction time—see Supplementary Fig. 6c–h). To more precisely quantify this, we plotted the reduction in d-prime as a function of reaction time, the latter quantified in control trials within that same session (as a proxy for behavioral reactivity in the same animals) (Fig. 5i, j, Supplementary Fig. 6i, j). Whereas early silencing invariably reduced performance, the effect of late silencing scaled with reaction time (Fig. 5j), and amounted to a complete impairment in visual detection (100% reduction) for sessions with the longest RTs. This impairment was not found for the animal’s propensity to lick (which affects false alarms and was quantified with the criterion parameter in our behavioral model; Supplementary Fig. 6k), suggesting that the effect of late inactivation on perceptual sensitivity increased as a function of rising RT. Furthermore, we considered the possibility that the reduction in d-prime relative to control trials was confounded by a lower d-prime on control trials for slow sessions, to begin with (Supplementary Fig. 2g, see also Supplementary Fig. 6l), but this did not account for the effect (Supplementary Table 1). Late silencing thus left performance intact in “fast” sessions in which hit/miss coding emerged quickly (mostly UST, but also some MST sessions) and reduced performance in slow sessions where hit/miss coding started after 200 ms (mostly MST sessions with higher cognitive demands, but note also how one slow UST session was affected; Fig. 5j).

### Causal involvement of late activity generalizes to visuotactile side detection

**Fig. 6:**
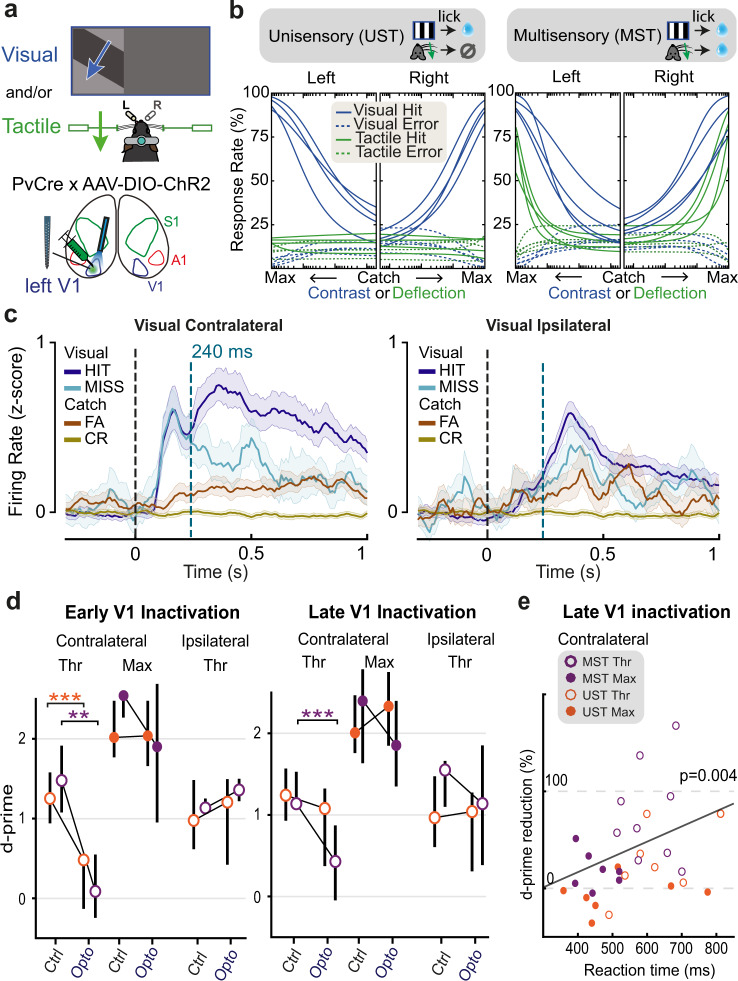
Extended causal requirement of V1 generalizes to visuotactile side detection. **a** Schematic of the visuotactile two-sided detection task in which mice reported the side of visual and/or tactile stimulation. **b** Psychometric fits for visual and tactile detection for each mouse trained in the UST and MST version of the task. Same conventions as in Fig. 1c–e, with the x-axis running left for visual/tactile stimuli presented to the left side and right for visual/tactile stimuli presented to the right side. Visual and tactile intensities were normalized for rendering purposes. **c** Average z-scored firing rate of responsive V1 neurons during visual (max contrast) and catch trials, split by trial outcome. Only neurons from MST mice are shown. The dashed blue line indicates the onset of optogenetic silencing. Similar to Task A, contralateral hits elicit more late activity compared to misses. Also note the weak early transient activity during FA trials (analogously to Task A, see Fig. 2e). Shaded area: bootstrapped 95% confidence intervals. **d** Visual d-prime across V1 inactivation conditions. Early V1 inactivation (left) impaired the detection performance of contralateral threshold-level visual stimuli in both UST (ANOVA, *n* = 7 sessions, *F*(1,14) = 24.57, *p* = 0.0006318) and MST (ANOVA, *n* = 6, *F*(1,12) = 17.93, *p* = 0.0023), without effect on ipsilateral threshold-level stimuli nor contralateral maximum-level stimuli. Late silencing (right) only affected detection performance of contralateral threshold stimuli in MST mice (ANOVA, *n* = 7 sessions, *F*(1,14) = 45.14, *p* = 0.000036), but not in UST mice (ANOVA, *n* = 7, *F*(1,14) = 2.15, *p* = 0.164). **p < 0.01, ***p < 0.001. Each corrected for four multiple comparisons (Bonferroni-Holm). Errorbars denote inter-quartile range. **e** Reduction in d-prime by late silencing correlated with the median reaction time on corresponding control trials (ANOVA, *n* = 30 conditions, *F*(1,26) = 9.785, *p* = 0.00427, *r*^2^ = 0.7056). Each dot represents a session.

So far, our results suggest that in the multisensory variant of the change detection task (MST), late V1 activity is causally involved whereas in the unisensory variant it mostly is not. However, UST and MST cohorts do not only differ by sensory contingencies, as UST mice were trained on a Go/No-Go paradigm, while MST mice learned a two-alternative choice task. Thus, the results we report could be due to differences in behavioral strategy rather than to changes in multisensory context. Furthermore, we wondered whether our results may extend to other sensory modalities. To address these aspects, we developed a visuotactile side detection task in which mice reported the side of sensory stimulation, i.e., instructing them to lick left for visual or tactile stimuli presented to the left and oppositely for the right side (Task B; Fig. 6a). Stimuli consisted of monocular drifting gratings (visual), whisker pad deflection (tactile), or a combination of both. In this task B, visual and tactile information need to be integrated as an inclusive-OR operation (rather than discriminated as in task A) to decide on which side (left/right) sensory stimuli appeared. Again, some mice were trained on responding only to vision to obtain reward (UST), while another cohort was trained on both vision and somatosensation (MST). Importantly, this UST version contained two response options and required responding to the correct lick spout (the visual stimulus could appear on the left or right). In addition to the differences with task A, this new task allowed us to test if our results extended to another multisensory processing principle (congruent combination of modalities instead of segregation^34^) and the detection of a different stimulus dimension (contrast instead of orientation change).

We controlled stimulus salience by varying visual contrast and whisker deflection amplitude and fitted the behavioral data with a psychometric model (Fig. 6b; see Methods). The visual detection threshold and task performance at maximum saliency were similar for both cohorts (UST and MST, Supplementary Fig. 8a, b). As in Task A, we found that visual reaction time (RT) decreased for higher stimulus saliencies (Supplementary Fig. 8c). In contrast with task A, however, RTs were similar between tactile and visual trials.

Pursuing the comparison with task A, laminar recordings in V1 revealed similar neural dynamics, with a marked early stimulus-driven component visible in contralateral visual trials, and late activity in both contra- and ipsilateral visual hits (Fig. 6c, Supplementary Fig. 8f; see also Supplementary Fig. 8g for lick-aligned neuronal activity). This late V1 activity was also present in tactile-only hits—so without visual stimuli—although only for MST mice (Supplementary Fig. 8h). We optogenetically silenced unilateral V1 either from stimulus onset (early silencing) or after a delay that separated the late from the early wave of activity (240 ms, late silencing; Fig. 6c). Early silencing of unilateral V1 reduced the detection of contralateral threshold-contrast stimuli in both UST and MST mice but spared detection of ipsilateral stimuli and full contrast stimuli (Fig. 6d). In MST mice, tactile detection was not affected by V1 silencing (Supplementary Fig. 8d). Consistently with our results for Task A, while early V1 silencing impaired detection of threshold-level visual stimuli for both unisensory and multisensory contexts, late V1 silencing only affected such detection in MST mice (Fig. 6d, Supplementary Fig. 8e). As in Task A, we observed that the effect of silencing increased for conditions with longer reaction time (Fig. 6e). Overall, results obtained with task B generalize our findings and confirm that the temporal window for the causal involvement of V1 in perceptual decision making is extended when subjects reinstate the more demanding, multisensory attentional set they have been trained on.

### Population decorrelation during hit trials precedes and is locked to reaction time

**Fig. 7:**
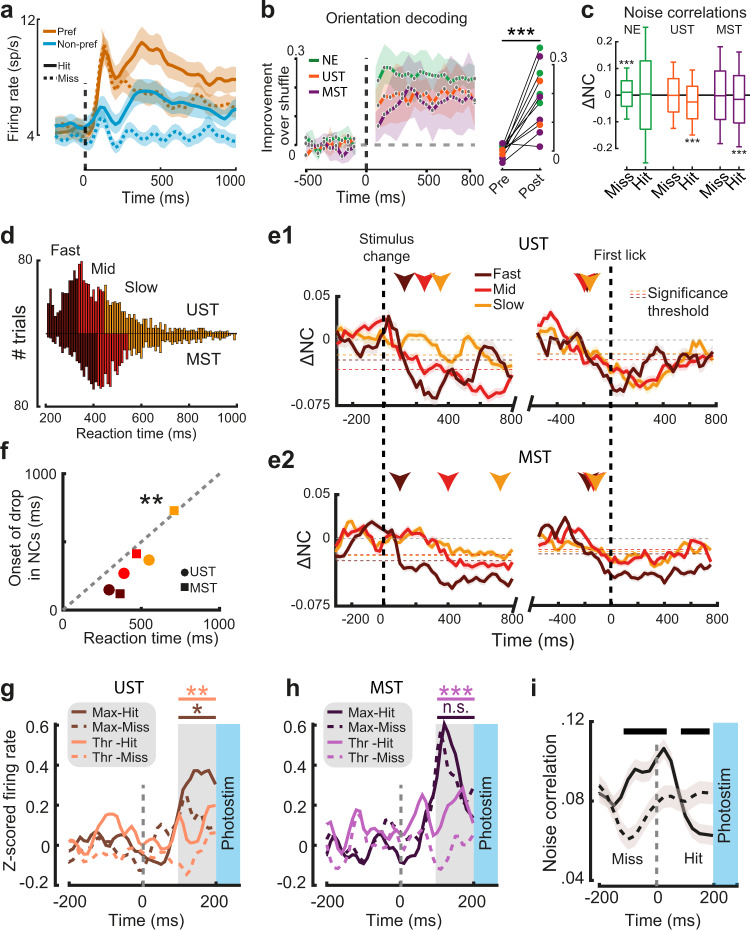
Onset of report-related activity in task A and drop in noise correlations predict effects of late silencing. **a** Average spiking rate for all orientation-selective neurons for preferred and non-preferred orientations is split by hits and misses (UST and MST neurons combined; task A). **b** Orientation decoding performance over time. Right panel: decoding performance increased post-stim (0 to +500 ms) versus pre-stim (−500 to 0 ms; *n* = 11 sessions, cohorts combined, ANOVA, *F*(1,17) = 44.76, *p* = 4.1 × 10^−6^) and increased in individual *sessions* from all cohorts (colored dots). **c** Change in noise correlation (NC) relative to baseline (200 to 1000 ms compared to baseline −500 to 0 ms) for visual trials split by choice and cohort (for auditory trials, see Supplementary Fig. 9a). Boxplots show the median and interquartile range (box limits) and 0.5 × interquartile range (whiskers). Noise correlations decreased only during hits in UST and MST mice (ANOVA, UST, *n* = 1930 pairs, *F*(1,3856) = 82.44, *p* < 1 × 10^−19^; MST *n* = 13972, *F*(1,28188) = 142.96, *p* < 1 × 10^−33^). Misses in NE mice were associated with a slight increase in noise correlations (*n* = 2904 pairs, *F*(1,5805) = 14.67, *p* < 0.001). **d** Reaction time distributions for visual hits in UST and MST cohorts and tertile ranges. **e1** Noise correlations over time with respect to baseline, either aligned to stimulus change (left) or first lick (right). Horizontal dashed lines indicate for each tertile the threshold for each tertile for the onset of the drop in NCs (below 2 standard deviations of the baseline; −500 to 0 ms) and this onset is highlighted with colored arrows. Note how noise correlations (aligned to stimulus change) drop first in fast trials, and progressively later in medium and slow trials. Right panels show that, when aligning to lick onset, the drop in noise correlations precedes reaction times by a similar lag, independent of reaction time tertile. **e2** Same as **e1**, but for MST mice. **f** Reaction time and moment of decorrelation were significantly correlated (Pearson correlation, *n* = 6, *r* = 0.960, *p* = 0.002). Scatterplot shows median reaction time and earliest time point of decorrelation for each tertile in the two visually trained cohorts. **g** Average Z-scored firing rates just before photostimulation (100–200 ms) were higher if the trial resulted in a visual hit rather than a miss in UST mice (Thr: *F*(1,156) = 10.16, *p* = 0.002; Max: *F*(1,152) = 5.66, *p* = 0.019; ANOVA). **h** Same as **g**, but for MST mice. Firing rates just before photostimulation were higher for hits than misses only for threshold visual changes (*F*(1,268) = 13.19, *p* = 0.001), but not maximal changes (*F*(1,254) = 0.59, *p* = 0.44). **i** Noise correlations for visual hit and miss trials before photostimulation onset (grouped across UST and MST cohorts and saliency levels). Black bar on top indicates time bins with significantly different NCs between hits and misses (*p* < 0.05). Throughout the figure, lines and shading are mean ± SEM.

The late report-related wave of activity during visual hits (Figs. 2e, 6c) likely arises through an interplay of higher-order areas that feedback to V1, possibly including premotor or other frontal areas^23,25,28,50^. The timing of this wave predicts the behavioral effects of late V1 inactivation, but the underlying mechanism governing the sculpting of a behavioral decision remains unclear. One possibility is that late activity is predominantly related to movement variables, coded orthogonally to sensory representations from a population perspective^26^. To investigate this, we further explored the properties of late activity. First, we tested whether the extended causal requirement of V1 was related to changes in the fidelity of sensory processing, as indexed by orientation decoding. For the audiovisual change detection task, we observed that, in line with the overall neuronal population (Fig. 2), orientation-selective neurons also showed a late hit-modulation of firing rate (Fig. 7a) and examined the effect of report-related activity modulation at a population level by training a random forest decoder to decode post-change grating orientation from V1 population activity. Orientation decoding was possible for hundreds of milliseconds after the orientation change with comparable performance across the three task versions (Fig. 7b). This suggests that in all visual trial types (regardless of task contingencies) information regarding the orientation of the stimulus was similarly present and that the extended requirement of V1 could rather be due to the interaction of this representation with the rest of the cortical circuit. Correlated firing rate fluctuations that are unrelated to signal coding (noise correlations—NCs) can impact information coding in populations of neurons^51–53^. NCs decrease as a function of various conditions, for instance when animals become experts at change detection^54^ or through attention^55^. We computed pairwise firing rate fluctuations over time for visual hits and misses. During baseline (−500 to 0 ms) NC values were comparable to the literature^56–59^ (0.063 ± 0.14 std) and decreased after stimulus change only during hits in UST and MST but not NE mice (Fig. 7c, Supplementary Fig. 9a). To investigate whether the onset of the decorrelation was related to reaction time, we split all visual hits from V1 recording sessions into three tertiles based on reaction time (Fig. 7d). Similar to behavioral data without recordings (Fig. 1h), UST mice reacted faster than MST mice (*p* = 0.0041, Wilcoxon rank-sum test). We quantified the earliest time-point where the drop in NCs reached significance (relative to baseline) for each tertile for UST and MST mice. NCs decreased at a latency that depended on reaction time, with the drop in NCs occurring later on slow compared to fast trials (Fig. 7e). The latency of the decrease in NCs and reaction time were significantly correlated (Fig. 7f), suggesting that population decorrelation is time-locked to reaction time. Indeed, noise correlations relative to the first lick (see Methods) dropped just preceding this first lick, irrespective of reaction time (Fig. 7e right part, Supplementary Fig. 9b), with the strongest decrease for visual hits in UST and MST mice and no decrease in surrogate hits in NE mice (Supplementary Fig. 9c). Overall, similarly to the onset of late, report-related activity, a late drop in NCs also precedes and is time-locked to perceptual report.

### Activity level and decorrelation predict the effect of late silencing

**Fig. 8:**
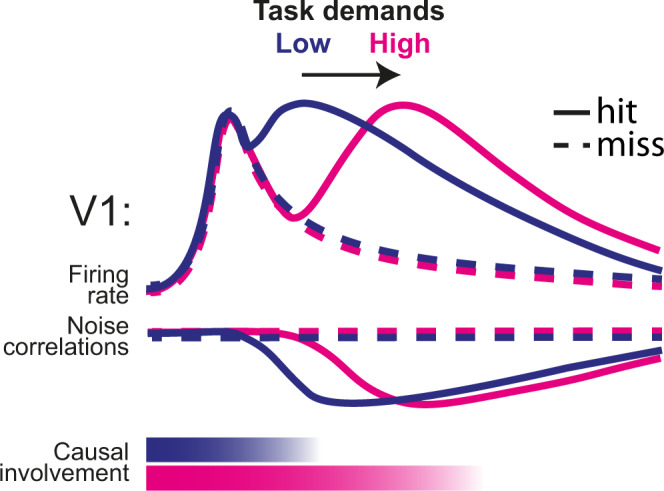
Schematic summary of results. Increased task demands (in our tasks imposed by multisensory requirements) delay the onset of the late report-related wave of activity and drop in noise correlations, and extend the causal involvement of V1. Jointly, these processes predict the behavioral effect of late V1 inactivation on visual detection, and whether a trial is going to be a hit or a miss.

If the late-onset increase in spiking activity and drop in NCs were related to perceptual report, one would expect that the variability in the behavioral effect of silencing (i.e., whether V1 inactivation was followed by a hit or miss) could be explained at the single-trial level by whether this drop had already occurred at the time of photostimulation. We, therefore, focused on V1 activity during visual trials just before late inactivation started (*n* = 230 cells), and tested whether the report-related firing-rate modulation and drop in NCs had both already occurred before 200 ms in hits but not misses. Indeed, hits were associated with increased activity just before photostimulation started (100–200 ms after stimulus onset) across levels of stimulus change and task versions (Fig. 7g, h). Similarly, NCs showed distinct profiles for hits and misses (Fig. 7i) and decreased just before silencing onset during hits but not misses. Surprisingly, NCs were higher just before and after stimulus change on hit trials (−125 to +25 ms around stimulus change), in contrast with^60^. Overall, these results show that increased firing rates, and the temporally coinciding drop of NCs in V1, correlate with and predict sensory report (Fig. 8).

## Discussion

In this study, we investigated the nature and function of late, recurrent activity in V1 in perceptual decision-making. An increase in multisensory task demands delayed behavioral decisions and extended the temporal window in which V1 was causally involved in determining perceptual report, given the same visual stimulus. As animals in the MST tasks were trained to process behaviorally relevant signals in two sensory modalities rather than one, longer reaction times (compared to UST tasks) are likely needed to integrate and compare information from distinct sensory modalities, also to assess which of two modalities is most likely to present an externally (as opposed to self-) induced sensory change. In particular, sensory selection was previously shown to affect neuronal processing already at the level of primary sensory cortices^61^, via a thalamocortical feedback loop^62^. Similarly, in our tasks the detection of a behaviorally relevant signal might modify how a given sensory modality is routed and processed^34^, leading to an extended requirement for V1 in multisensory contexts. Furthermore, in our experiment, a specific visual stimulus under conditions of low saliency (i.e., a small change in grating orientation) required more time to determine whether there was a change than in the case of high saliency (Fig. 1h, f). Therefore, our results may not be specific to multisensory contexts (Fig. 5j). Indeed, increased cognitive demands (implemented in terms of memory-dependent vs. visually-guided navigation) were shown to broaden the contribution of dorsal cortical areas to perceptual decision-making beyond V1^63^. In conditions of high task demands, these and the present results suggest that higher-order cortices may need to rely on the output of V1 for longer temporal windows compared to simpler (e.g., purely visually guided) tasks. Importantly, we obtained similar results in two completely independent experimental groups (Task A and B). This further supports the existence of a link between task demands and temporal extension of V1 requirement.

We found modest differences in onset latencies and orientation coding of visually evoked V1 responses across task contingencies and cohorts, suggesting that the dynamics of bottom-up, feedforward processing are mostly conserved. In contrast, striking differences were found in the late phase of V1 activity, and particularly in the behavioral consequences of late optogenetic inactivation. Previous studies in humans and non-human primates showed that attention-related activity in V1, similarly to report-related activity, emerges in a late phase of V1 sensory-evoked responses as a consequence of top-down modulation^13–16,21^. A potential role of attention-related V1 activity in perceptual decision-making—and not only for processing visual stimuli—was also suggested^64^. Report-related late activity is also thought to arise from recurrent feedback emitted by higher-order cortices^37^, in agreement with the predominance of report-related coding in deeper cortical layers (Fig. 4c, see also Fig. 2f). In our experiments, silencing late V1 activity abolished the detection of orientation changes and contralateral stimuli in conditions of high cognitive demands (and consequently long reaction time), as is the case when having to process multiple sensory modalities simultaneously. This demonstrates a causal role of late V1 activity in perceptual decision making which is independent of scene complexity (cf.^22^). However, this result is in apparent conflict with recent studies, which showed that late inactivation of V1 did not affect perceptual decisions^31^, unless stimuli to be processed became more complex^22^. A possible explanation lies in the fact that, for relatively simple tasks, the window of temporal involvement of V1 in perceptual decision making might overlap with early, sensory-evoked activity. In line with this, the effectiveness of late inactivation was most prominent for low-saliency stimuli in MST mice and linearly scaled with reaction time (Figs. 5j, 6e). The only exception was the detection of high contrast stimuli in MST (but also UST) mice in task B, which was affected neither by early nor late V1 silencing (Fig. 6d, e). This suggests that subcortical structures (e.g., superior colliculus) may suffice to localize highly salient stimuli^65^ in task B (in contrast to task A, which requires detecting an orientation change), although we cannot fully exclude that portions of V1 were not completely inactivated. Furthermore, it is unlikely that late V1 silencing generally impaired cortical network processing^66^, as it did not affect ipsilateral visual detection in the visuotactile detection task (task B; Fig. 6), nor tactile or auditory performance (Supplementary Fig. 6b, Supplementary Fig. 8d). A final point relates to the increase in FAs following inactivation of late V1 activity in Task A (Fig. 5f, Supplementary Fig. 6f), which might suggest a different pathway for the observed behavioral effects. First, the criterion parameter of the psychometric model indicated that the behavioral effect of late V1 inactivation was not driven by this increase in FAs. Second, no increase in FAs was found in task B, which indicates that this increase may be specific to task A, in which mice were trained to react to any change in visual stimuli. Indeed, inactivation of V1 might be interpreted as a change in visual scene or view, as indicated by the fact that V1 inactivation did not increase FAs during auditory trials; moreover, no increase in FAs was observed in the control inactivation of S1 (Supplementary Fig. 7).

We optogenetically silenced both sensory and report-related components of V1 activity, which are jointly present in the late window (Fig. 3c). Importantly, late V1 inactivation in the absence of an early sensory-related component (e.g., ipsilateral visual stimuli in task B, or non-visual hit trials) did not impair behavioral responses, in agreement with a recent study suggesting that the report-related component alone is not sufficient for perceptual task performance^22,49^. However, the question remains how sensory-evoked and report-related activity are related to each other during the late phase^49^. On the one hand, increased task demands may prolong sensory processing of the visual stimulus, or at least the time downstream regions need to sample the ongoing flow of V1 activity to gather sufficient evidence on visual stimulus change^67,68^. On the other hand, recurrent interactions between visual cortex and connected regions during late windows may jointly influence sensory representation, in line with the predictive processing framework^19,37,69^. In other words, in conditions requiring extended processing time, V1 and downstream regions need to interact for longer periods to jointly construct a behaviorally conclusive representation of the modality-specific change (task A) or the side of stimulus presentation (task B). The co-occurring increase in firing rate and drop in NCs that precede reaction time in hit trials likely originates in the pre-motor cortex, as previously suggested^23^. However, the question remains whether the behavioral effect of late V1 inactivation is due to a prolonged window during which downstream regions process information coming from V1, or rather if the late-onset report-related activity and the related drop in NCs recursively interact with ongoing sensory processing^50,69,70^ to shape perceptual decision making. Further experiments will be required to fully understand the mechanisms linking late V1 activity to perceptual report.

In conclusion, our results show that, although all sensory information that is theoretically required to perform a task is available in V1 shortly after stimulus onset, transforming such sensory inputs into a perceptual representation requires substantial recurrent interplay between cortical areas, which is temporally extended by factors increasing task demands (such as multisensory interactions). Our results thus dispute the classical picture of perceptual decision making: late-onset activity in primary visual cortex, which primarily stems from cortico-cortical recurrent interactions^22,71,72^, is not simply involved in relaying, refining, and modulating the processing of complex visual stimuli, but also provides a causally relevant temporal window for perceptual decision-making.

## Methods

### Materials availability

The study did not generate any unique reagents.

### Experimental subjects

All animal experiments were performed according to national and institutional regulations. The experimental protocol was approved by the Dutch Commission for Animal Experiments and by the Animal Welfare Body of the University of Amsterdam. We used two transgenic mouse lines: PVcre (B6;129P2-Pvalbtm1(cre)Arbr/J, RRID: IMSR_JAX:008069) and F1 offspring of this PVcre line and Ai9-TdTomato cre reporter mice (Gt(ROSA)26Sor^tm9(CAG-tdTomato)Hze^ RRID: ISMR_JAX 007909). A total of 49 male mice were used for this study:

- Task A: 28 mice (NE group: 7 mice, UST group: 4 mice, MST group: 17 mice)
- Task B: 12 mice (UST group: 4 mice, MST group: 8 mice)

Littermates were always assigned to the same experimental group. Mice in our colony are backcrossed to C57BL/6J wild-type mice from Jackson Laboratories every ten generations. Mice were at least 8 weeks of age at the start of experiments. Mice were group-housed in a pathogen-free facility under a reversed day-night schedule (lights were switched off at 8:00 and back on at 20:00). All experimental procedures were performed during the dark period. Temperature in the housing facility was maintained between 19.5 and 23.5 °C, and humidity was kept in a range between 45 and 65%. This study did not involve randomization or blinding. We did not predetermine the sample size. Some subjects were unable to successfully learn to make decisions based on both modalities (MST task versions) within 2 months and were excluded from further experiments. Although experiments were performed in male mice only, recent studies suggest that no difference should be expected in female mice as concerns the neuron-level mechanisms of perceptual decision making—see e.g., refs. ^22,27^.

### Head-bar surgery

Before the start of any experiments, mice were implanted with a headbar to allow head-fixation. Mice were subcutaneously injected with the analgesic buprenorphine (0.025 mg/kg) and maintained under isoflurane anesthesia (induction at 3%, maintenance at 1.5–2%) during surgery. The skull was exposed and one of two types of custom-made titanium circular head-bars with a recording chamber (version 1: inner diameter 5 mm, version 2: inner diameter 10 mm) was positioned over the exposed skull to include V1 and attached using C&B Super-Bond (Sun Medical, Japan) and dental cement. For task A in which visual stimuli were centrally presented binocular V1 was targeted based on the following coordinates (relative to lambda): AP 0.0, ML+/− 3.0^73^. Whereas coordinates sufficed for task A, for task B, in which lateralized visual stimuli were used, V1 was targeted using intrinsic optical imaging (see below) to localize the retinotopic region of V1 corresponding to the region of visual space in which the lateralized visual stimuli were presented. The skin surrounding the implant was covered using tissue adhesive (3 M Vetbond, Maplewood, MN, United States) to prevent post-surgical infections. The recording chamber was covered with silicon elastomer (Picodent Twinsil). Mice were allowed to recover for 2–7 days after implantation, then habituated to handling and head-fixation before starting on the training procedure.

### Behavioral training

Mice were subjected to a water restriction schedule and minimum weight was kept above 85% of their average weight between P60-P90. They typically earned their daily ration of liquid by performing the behavioral task but received a supplement when the earned amount was below a minimum of 0.025 ml/g body weight per 24 h. Mice received ad libitum food.

Mice were head-fixed in a custom-built headbar holder in a dark and sound-attenuated cabinet. The body of the mouse was put in a small tube to limit body movements. The task was controlled in Octave (GNU Octave 4.x) interfacing with Arduino microcontroller boards (Arduino Uno, with code compiled in Arduino IDE 1.0.8). Licks were detected by capacitance-based or piezo-electric-based detectors. Upon correct licking, i.e., in hit trials, 5–8 μl of liquid reward (infant formula) was delivered immediately using gravitational force and solenoid pinch valves (Biochem Fluidics). Reward volume was calibrated biweekly to prevent lateralized response bias due to unequal reward size.

### Behavioral task A: audiovisual change detection

Auditory and visual stimuli were continuously presented throughout a behavioral session. During visual trials a feature changed (the orientation of a drifting grating), after which the visual display of this altered feature continued (post-change orientation) until the next visual change, and similarly for the auditory stimuli (post-change frequency).

#### Visual stimuli

Visual stimuli consisted of full-field drifting square-wave gratings that were continuously presented with a 60 Hz refresh rate on an 18.5-inch monitor positioned at a straight angle with the body axis from the mouse at 21 cm from the eyes. Gratings were presented with a temporal frequency of 1.5 Hz and spatial frequency of 0.08 cycles per degree at 70% contrast and gamma-corrected. In trials with a visual change the orientation of the drifting grating was instantaneously changed (e.g., from 90° to 120°) while preserving the phase. The degree of orientation change determined the visual saliency and was varied across experimental conditions.

#### Auditory stimuli

Each auditory stimulus was a combination of five pure tones at harmonic frequencies. It was composed mainly of a center tone, as well as two lower and two higher harmonics (octaves below and above the center tone). If *f*_0_ is the center tone: *f*_−2_ = ¼*f_0_*, f*_−1_ = ½*f_0_*, f*_0 *=*_ *f*_0_*; f*_+1_ = 2*f_0_; *f*_+2_ = 4*f_0_. All frequencies were expressed in scientific pitch as powers of 2 with the center tones spanning from 2^13 ^Hz (=8372 Hz) to 2^14 ^Hz (=16,744 Hz). An example stimulus, 2^13.5^ (named by center tone), was therefore composed of five pure tones of 2^11.5^, 2^12.5^, 2^13.5^, 2^14.5^, and 2^15.5^ Hz. The weight with which each tone was present was taken from a Gaussian distribution across all tones for all stimuli, centered at 2^13.5^ (=11,585 Hz). Because of this fixed weight distribution, stimuli with higher center tone frequency have decreasing weights for higher harmonics and increasing weights for lower harmonics. Stimuli with higher center frequency are thus increasingly made up of lower frequency components to the point of arriving at the starting stimulus (see also Supplementary Fig. 1). This auditory stimulus design with harmonics and fixed weights was inspired by the Shepard tone illusion^39^. However, in contrast to this illusion, our stimuli were static and not sweeping across frequencies, and the original illusory aspect of a tone ever-increasing (or decreasing) in pitch was not exploited. The primary reason for this auditory stimulus design was the circular nature of the stimulus set, which mirrored the visual stimulus set with drifting gratings in all orientations.

During auditory trials, one stimulus was changed instantaneously to another, resulting in a shift in spectral power to five new frequencies. Auditory changes were expressed as partial octaves, with ½ octave maximally salient and the minimal change used was 1/256 partial octave. The degree of frequency/octave change determined the auditory saliency and was varied across experimental conditions. During auditory stimulus changes, the phase across all tones was preserved. Stimuli were presented with a sampling rate of 192 kHz. Stimuli were high-pass filtered (Beyma F100, Crossover Frequency 5–7 kHz) and delivered through two bullet tweeters (300 Watt) directly below the screen. Note that this high-pass filter eliminated the lowest frequency components of the Shepard stimuli, and left the mid and high frequency components intact (those that span the sensitive part of the mouse hearing range, 8–16 kHz). This was done to prevent damage to the specialized tweeters that we used, but did not affect the animals’ ability to report even very small differences between subsequently presented Shepard tones. Sound pressure level was calibrated at the position of the mouse and volume was adjusted per mouse to the minimum volume that maximized performance (average ± 70 dB).

In an earlier cohort of mice trained on task A (*N* = 13/28), the Shepard tones (1) were expressed in absolute Hz (e.g., an auditory trial with Δ2 kHz changed from 8 kHz to 10 kHz), (2) had 9 harmonics, (3) were presented with a sampling rate of 48 kHz and (4) were not phase-preserved during a change in auditory frequency. We observed no qualitative or quantitative differences in both neural and behavioral results between the cohorts (behavior between cohorts is compared in Supplementary Fig. 2). The horizontal axes were normalized in Fig. 1 to accommodate all mice.

With both auditory and visual stimulus sets being circular, the direction of change (clockwise or anticlockwise) was behaviorally irrelevant (isotropy), and the only relevant dimension was the amount of change. Given the use of the full auditory spectrum and full-field visual gratings, stimuli in both modalities allowed change detection based on feature selectivity while recruiting neurons across the tonotopic organization of auditory cortex^74^ and across the retinotopic map of visual cortex - which in our case benefitted both neural data acquisition and interventions.

#### Versions of Task A

Animals were assigned to one of three versions of a change detection task (NE, UST, MST) in which the visual and auditory stimuli were identical and only the reward contingencies varied. As we performed additional experiments with animals from the MST cohort, this resulted in a higher number of animals in the MST cohort.

*NE*: *Noncontingent exposure* (*N* = 7/28 animals)—In this version, neither modality was associated with reward availability. Both the auditory and visual stimuli were continuously presented with the same distribution of trial types and temporal statistics as the multisensory version (see below). To compare intermittent licks, rewards, and stimuli across task versions, we sought to achieve similar rates of licking and reward delivery. Therefore, mice in this version could obtain rewards in a hidden “response window” (a 1500 ms time interval in which either left or right licks could be emitted to acquire reward; same duration as MST, below). This response window was temporally decorrelated from the stimuli. Mice thus licked spontaneously at the two spouts and received occasional rewards. Mice were exposed 2–5 days to this behavioral task before any experiments.

*UST*: *Unisensory version* (*N* = 4/28 animals)—In this version, only visual change was associated with reward availability. Mice were trained to respond to the visual changes only. Continuous auditory stimuli and changes were presented throughout training and recording sessions with the same statistics as the multisensory version, but were not associated with reward and were temporally decorrelated from the task-relevant visual trials. Given that only one side was rewarded in this version, spontaneous licking to this side had a higher probability of being rewarded and therefore the response window was shortened to 1000 ms (i.e., in this window, licks could be produced to acquire reward).

*MST*: *Multisensory version* (*N* = 17/28 animals)—In this version visual and auditory change were both associated with reward availability. Mice were trained to respond in a lateralized manner to each modality: lick to one side to report visual changes, to the other side in case of auditory changes (modality-side pairing was counterbalanced across mice). Therefore, in this version, subjects had to simultaneously monitor both the auditory and visual modality, detect changes in a feature and discriminate the modality in which the change occurred. In other words, mice were required to identify the sensory modality in which a change occurred.

#### Training stages

For each trained modality (vision in UST, vision and audition in MST), training occurred in steps. In the first stage learning was facilitated by (1) only including the easiest trial type (maximally salient trials: 90° orientation change for the visual domain^75^ and 4 kHz or ½ octave—in earlier and later cohorts, respectively—for the auditory domain), (2) additional instantaneous changes to increase saliency, (3) a passive reward on the correct side if the animal did not respond within 900 ms, and (4) the opportunity to correct after choosing the incorrect side. These facilitating conditions were phased out throughout the training procedure and trials of varying lower saliency were introduced. Animals were trained until their psychometric curve in the target modalities reached a plateau. For the MST version, animals were first trained in one modality, then the other, after which they were combined (the order of modalities was counterbalanced across mice).

Trials types were pseudorandomly presented (block-shuffled per 10 trials, 10% of trials were catch trials, thus without a stimulus change, 41% visual trials, 41% auditory trials, 8% multimodal trials—see below). The inter-trial interval was taken randomly from an exponential distribution with a mean of six seconds (minimum 3 and maximum 20 s). Directly after a stimulus change, a response window of 1500 ms followed in which either left or right licks could be emitted to acquire a reward. Licks during the first 100 ms were not counted as these occurred too early to be considered part of a stimulus-response sequence. The first lick after this “grace period” was registered as the animal’s choice and correct licks were directly rewarded. To counter any bias in MST mice, if the fraction of licks to one spout out of all licks in the last 10 trials was above 90%, the next trial was selected with a 95% probability to be of the other modality. As visual and auditory feature changes were associated with conflicting motor actions (only in the multisensory version of the task), a multimodal trial (simultaneous audiovisual change) would present the animal with conflicting signals. We introduced these conflict trials in a subset of sessions, but these trials were not included in the current analyses.

For each trained animal (before any recordings) we implemented three behavioral sessions in which we presented five levels of auditory and visual saliency that spanned the perceptual range to establish the perceptual sensitivity of each mouse. We fit the concatenated data of these three sessions with a cumulative normal distribution per modality with four free parameters^76^:

1 $$f\left(x\right)={{{{{\rm{\gamma }}}}}}+\left(1-{{{{{\rm{\gamma }}}}}}-{{{{{\rm{\lambda }}}}}}\right)\left(\frac{1}{2}\left[1+{{{{{\rm{erf}}}}}}\frac{x-{{{{{\rm{\mu }}}}}}}{{{{{{\rm{\sigma }}}}}}\sqrt{2}}\right]\right)$$

Here, *γ* describes the false alarm rate (spontaneous licks during catch trials), *λ* the lapse rate (misses at maximal saliency), *μ* the mean (perceptual threshold), and *σ* the standard deviation (sensitivity to variations of stimulus intensity). Having established the psychometric function per mouse, we took four levels of saliency per modality at fixed points along the psychometric function: subthreshold (*μ* − *σ*; *sub*), threshold (*μ*; *thr*), suprathreshold (*μ* + *σ*; *sup*), and maximal saliency (*max*). The visual threshold ranged from 4° to 12°, and the auditory threshold from 10–100 Hz (frequency version) or 1/64–1/16 partial octave (octave version) (Supplementary Fig. 2). This analysis was purely performed to select stimulus intensities of equal subjective saliency across mice for the experiments. All other analyses were based on fitting the behavioral data with a psychometric signal detection model (see below).

In recording sessions, we limited conditions to sample sufficient trials per modality × feature × saliency × choice combination. First, we only used two levels of change: threshold and maximal saliency. For NE mice and auditory conditions in UST mice, we used threshold values that matched those from trained animals. Second, we only used four orientations or tones. Specifically, this means that stimuli jumped between A, B, C, and D, where distance AB and CD are around threshold and distance AC and BD are maximal. An example stimulus set for a mouse with a visual threshold of 7˚ and an auditory threshold of 1/32 octave was therefore for the visual domain: *A* = 100˚, *B* = 107˚, *C* = 190˚, *D* = 197˚, and for the auditory domain (in Hz): *A* = 2^13.25^, *B* = 2^13.25+1/32^, *C* = 2^13.75^, *D* = 2^13.75+1/32^.

### Behavioral task B: visuotactile side detection

As in paradigm A, mice were trained on one of two versions of a visuotactile detection task: a multisensory version, where both visual and tactile modalities were informative on the side that needed to be chosen to acquire reward (MST) and a unisensory version, where the tactile modality was present as well, but only the visual modality was informative (UST).

#### Stimuli used

##### *Visual stimuli*

Visual stimuli consisted of square-wave drifting gratings, with a temporal frequency of 1.5 Hz, a spatial frequency of 0.025 cycles per degree and 30° orientation. The contrast of the gratings was modulated per trial to control detection difficulty. Visual stimuli were generated in Octave using Psychtoolbox3 and were presented monocularly at >24° from azimuth on each side^77^ with a gamma-corrected 18.5-in. monitor at a frame rate of 60 Hz and a distance to the eye of 18 cm.

##### *Tactile stimuli*

Tactile stimuli consisted of a single deflection of the whisker pad using a piezoelectric bender (PL128.10, Physik Instrumente) coupled to a 5 cm long pipette ending on a 5 × 5 mm patch of Velcro. A voltage driver (E650, Physik Instrumente) and an RC filter were used to produce a backward deflection of the bender with an exponentially decaying speed (*τ* = 72 ms) during 360 ms, followed by a forward deflection with the same characteristics. The amplitude of the deflection was modulated to control detection difficulty. Elicited whisker deflection angles ranged from 0° to 3.6°. For both visual and tactile stimuli, stimulus intensity was adjusted individually to match the desired saliency.

#### Versions of task B

##### *UST: Unisensory version*

Visual and/or tactile stimuli were presented to either the right or left side of the animal. To obtain a reward, mice had to detect the side where the visual stimulus was presented and lick the spout at the corresponding side. In this version, only the visual modality was informative on reward availability. Tactile stimuli were delivered but not associated with reward and tactile and visual stimulus sides were decorrelated. The inter-trial interval was drawn from an exponential probability distribution with a mean of 4 s (minimum 3, maximum 7; with a 22% chance of catch trial (no stimulus, no reward) and a maximum of two catch trials in a row, a mouse could wait up to 21 s before another stimulus was displayed). Visual and/or tactile stimuli were presented for 1 s. In a multisensory trial (not analyzed here), the tactile stimulus was presented with a lag of 70 ms after the visual stimulus onset (similar to ref. ^47^). Licks were only rewarded in the interval of 140–1000 ms after stimulus onset. While a correct lick triggered reward delivery, an incorrect lick (i.e., to the wrong side) terminated the trial and aborted stimulus presentation. Trials were pseudo-randomly generated by blocks of 60 with 22% catch trials, 12% tactile-only trials, 53% visual-only trials, and 13% multisensory trials.

##### *MST: Multisensory version*

In this version, both visual and tactile modalities were informative on reward availability. In multisensory trials, visual and tactile stimuli were presented on the same side. Overall, task B required the mouse to follow an Inclusive-Or rule (lick to the side with either a visual or tactile stimulus, or a compound stimulus in both modalities). During training, mice first learned to detect tactile stimuli. Multisensory trials were then added and finally, visual-only trials were introduced so that mice could eventually detect visual and/or tactile modalities. Since tactile trials were rewarded, to keep the reward/no-reward balance, we increased the number of tactile trials: 25% catch, 25% visual-only, 25% tactile-only, 25% multisensory. Otherwise, both unisensory and multisensory task versions had the same parameters.

The noncontingent exposure (NE) version was not implemented for task B.

### Imaging, optogenetics, and electrophysiology

#### Intrinsic optical imaging

To localize the primary visual cortex in task B experiments, we performed intrinsic optical imaging (IOI) under lightly anesthetized conditions (0.7–1.2% isoflurane). A vasculature image was acquired under white light before starting the imaging session. During IOI, the cortex was illuminated with 630 nm light and images were acquired using a CCD camera connected with a frame grabber (Imager 3001, Optical Imaging Inc, Germantown, NY, USA), defocused about 500 µm below the pial surface. Visual stimulation consisted of square-wave drifting gratings (duration 8 s, 2 Hz, 0.05 cycles/deg, 100% contrast) presented in the right visual hemifield. All image frames obtained during stimulus presentations were divided by the average of the first 10 frames acquired just before stimulus presentation. The target area was outlined as the region with visually-evoked decrease in reflectance, using custom-made software in MATLAB^47^.

#### Viral injection

We performed viral injections and optogenetic experiments in a total of 25 mice, representing a subset of the full experimental cohort (Task A: *n* = 4/4 UST, *n* = 9/17 MST; Task B: *n* = 4/4 UST, *n* = 4/8 MST). Mice were subcutaneously injected with the analgesic buprenorphine (0.025 mg/kg) and maintained under isoflurane anesthesia (induction at 3%, maintenance at 1.5–2%) during surgery. We performed small craniotomies (±100 μm) over V1 using an ultrafine dental drill and inserted a glass pipette backfilled with AAV2.1-EF1a-double floxed-hChR2(H134R)-EYFP-WPRE-HGHpA (titer: 7 × 10¹² vg/mL, 20298-AAV1 Addgene). In total 50 nL was injected in V1 (bilateral binocular V1 for Task A and unilateral V1 for Task B) at 700 μm and 400 μm below the dura (25 nL per depth) using a Nanoject pressure injection system (Drummond Scientific Company, USA).

#### Optogenetics

In a random subset of trials (50% of trials for task A, 25% for task B) photostimulation started at stimulus onset (early inactivation) or was delayed (late inactivation). For the MST version of task B, early and late inactivation took place in separate sessions. Late inactivation occurred after 200 ms in Task A and 240 ms in Task B. Photostimulation continued until the animal made a choice. We interleaved sessions in which we positioned the fiber over V1 with control sessions in which we either positioned the optic fiber over area S1 (where no virus was injected) or at the head-bar. To locally photostimulate V1, a 473 nm laser (Eksma Optics, DPSS 473 nm H300) was connected to one or two fiber-optic cannulas (ID 200 um, NA 0.48, DORIC lenses) that were positioned directly over the thinned skull at the area of interest (bilateral V1 for Task A and unilateral V1 in Task B). Light delivery was controlled by a shutter (Vincent Associates LS6 Uniblitz) with variable pulse and interpulse duration with an average of 20 Hz and 75% duty cycle (Task A) or with 10 ms pulses sequentially interleaved by 20 ms and 30 ms (~72% duty cycle, Task B). The shutter was located in a sound-insulated box distal from the experimental setup. As we simultaneously performed extracellular recordings in V1 of all mice, we adjusted laser power for each animal to the minimum power that maximally inhibited neural activity. This was commonly 2–15 mW at the tip of the each fiber (placed 0.5–2 mm above the cortical surface) corresponding to an effective 1.5–5.25 mW/mm^2^ at the cortical surface (taking the ~75% duty cycle into account), which is below the levels that produce unwanted heating in tissue^22,78^.

To prevent light from reaching the eye of the mouse, the cannulae were sealed with black tape, leaving only the tip exposed. Furthermore, sessions with optogenetic manipulation were performed in an environment with ambient blue light. Even though we implemented these measures, we observed an increase in false alarms in some mice in task A. This suggests either that mice could perceive the laser, or that our manipulation evoked perceptual changes that were reported as a trial. We therefore verified (1) that our main effect of late silencing was not explained by a change in criterion (see Behavioral Analysis Task A), (2) positioned the fiber over uninfected somatosensory cortex (S1), and (3) performed the same optogenetic experiments in a second visuotactile paradigm where we did not have an increase in False Alarm responses by photoinactivation of V1. In Task B, due to a lack of sufficient trials to test optogenetic silencing for all conditions, only the reported conditions were tested (Visual Contralateral Thr and Max, Visual Ipsilateral Thr, Tactile Contralateral Thr) as well as Multisensory Contralateral Thr.

#### Extracellular recordings

Mice were subcutaneously injected with the analgesic buprenorphine (0.025 mg/kg) and maintained under isoflurane anesthesia (induction at 3%, maintenance at 1.5–2%) during surgery. We performed small (about 200 μm) craniotomies over the areas of interest (up to 6 per animal) using a dental drill. The recording chamber was sealed off with silicon elastomer and the mice were allowed to recover for 24 h.

Extracellular recordings were performed on consecutive days with a maximum of 4 days to minimize damage to the cortex. Microelectrode silicon probes (NeuroNexus, Ann Arbor, MI—four types of either 32 or 64 channels were used, catalog numbers A1 × 32-Poly2–10 mm-50 s-177, A2 × 16-10 mm-100-500-177, A4 × 8-5 mm-100-200-177, A1 × 64-Poly2-6 mm-23 s-160) were slowly inserted in the cortex until all recording sites were in contact with the tissue. V1 was approached perpendicularly to the cortical surface. The medial prefrontal cortex, primary auditory cortex, and posterior parietal cortex were also recorded, but data from these areas were not analyzed here. After insertion, the exposed cortex and skull were covered with 1.3–1.5% agarose in artificial CSF (125 mm NaCl, 5 mm KCl, 1.3 mm MgSO_4_, 2.0 mm NaH_2_PO_4_, 2.5 mm CaCl_2_, pH 7.3) to prevent drying and to help maintain mechanical stability. The probe was left in place for at least 15 min before recording to allow for tissue stabilization. Electrodes were dipped in DiI (ThermoFisher Scientific) during the final recording session allowing better post hoc visualization of the electrode tract. The ground was connected to the head bar and the reference electrode to the agarose solution. Neurophysiological signals were pre-amplified, bandpass filtered (0.1 Hz to 9 kHz), and acquired continuously at 32 kHz with a Digital Lynx SX 64/128 channel system in combination with the acquisition software Cheetah 5.0 (Neuralynx, Bozeman, MT).

Spike sorting of data acquired during task B was done with custom-made software in MATLAB, as previously described^47^, and only units having less than 1% of their spikes within a 1.5 ms refractory period were kept. For task A we used Klusta 3.0.16 and then manually curated with the Phy GUI^79^ (Phy 1.0.9). Before spike sorting the median of the raw trace of nearby channels (within 400 μm) was subtracted to remove common artifacts. Each candidate single unit was inspected during manual curation based on its waveform, autocorrelation function, and its firing pattern across channels and time. Only high-quality single units were included, defined as having (1) an isolation distance higher than 10 (cf. ref. ^80^) (2) less than 0.1% of their spikes within the refractory period of 1.5 ms^81,82^, (3) spiking present throughout the session. Neurons were deemed stably present if they had spikes in more than 90 out of 100 time bins during the entire session.

#### Recording depth estimation

The estimation of the laminar depth of the electrodes in V1 was based on three aspects. First, we computed the power in the 500–5000 Hz range to localize layer 5 with the highest MUA spiking power^83^. Second, we showed contrast-reversing checkerboards before each recording session and computed the current source density profile to estimate layer 4 with the earliest current sink, as previously described^84^. Lastly, this was aligned with the depth registered when the silicon probes were lowered from the dura. The granular layer was taken to span from 400 to 550 μm from the dura.

#### Video monitoring

In Task A, the left eye (ipsilateral to the hemisphere of recording) was illuminated with an off-axis infrared light source (six infrared LEDs 850 nm) adjusted in intensity and position to yield high contrast illumination of both the eye and whisker pad. A frame-grabber acquired images of 752 × 582 pixels at 25 frames per second through a near-infrared monochrome camera (CV-A50 IR, JAI) coupled with a zoom lens (Navitar 50 mmF/2.8 2/3” 10MP) that was positioned at ~30 cm from the mouse.

To extract pupil variables^59,85^ we trained DeepLabCut^86^ (version 2.1.10) on 300 frames from 15 video excerpts of 1–2 min with varying pupil size, illumination, contrast, imaging angle, and task conditions. We labeled the pupil center and six radially symmetric points on the edge of the pupil. An ellipsoid was fit to these six outer points. The pupil center was taken as the center of the ellipsoid and the pupil area as the ellipsoid area from the fitted ellipse parameters. Single poorly fit frames were replaced by the running median (10 frames). We z-scored the total session trace.

#### Histology

At the end of each experiment, mice were overdosed with pentobarbital and perfused (4% paraformaldehyde in phosphate-buffered saline), and their brains were recovered for histology to verify viral expression and placement of silicon probes in V1. We cut coronal 50 µm sections with a vibratome, stained them with DAPI, and imaged the mounted sections. For flattened cortical sections (e.g., Fig. 5b) we first removed subcortical tissue and flattened the cortical sheet of each hemisphere between glass slides by applying pressure overnight before sectioning 100 µm slices with the vibratome^87^. For coronal sections, area borders were drawn by aligning and overlaying the reference section from the atlas^88^. For flattened cortical sections, area borders were drawn based on cell densities aligned to reference maps^89^.

We frequently observed a minor reduction in fluorescence at the center of the viral injection site. Therefore, a potential concern could be that the cortical circuit at the center of the site could be either affected in function or less responsive to optogenetic inactivation. However, visual task performance was similar before and after viral injection (d-prime around 1.5 in maximal visual saliency control trials; Figs. 1f, 5g). Moreover, optogenetic inactivation was highly effective in silencing pyramidal cell activity and in impairing visual discrimination for difficult visual trial types during both behavioral tasks (Figs. 5g, 6d).

### Quantification and statistical analysis

#### Data analysis

Unless otherwise stated, all data were analyzed using custom-made software written in MATLAB (R2016a, The MathWorks, Natick, MA).

#### Behavioral analysis—Task A

Sessions were terminated when the animal did not respond for 20 trials and these last 20 trials were discarded from analyses. Sessions in which the hit rate for maximal auditory and visual changes was below 30% were excluded.

Behavioral response rates in task A were fit with a multi-alternative signal detection model^40^. This model extends signal detection theory^45^ and aims to accurately and parsimoniously account for observer behavior in a detection task with multiple signals. In this model, the decision is based on a bivariate decision variable whose components encode sensory evidence in each modality. Decision space is partitioned into three regions (no response: neither evidence is strong enough; auditory response, and visual response). In a given trial, the observer chooses to report visual or auditory stimuli if the decision variable exceeds a particular cutoff value, the “criterion” for each signal (the animal’s internal signal threshold for responding, in terms of signal detection framework). We fit two versions of this model. In sessions with two levels of saliency (threshold and maximum), we fit the d-prime (*d*′) and criterion (*c*) to the behavioral response rates separately for each stimulus change intensity. This consists of fitting four free parameters (*d*′ and *c* for each modality). In sessions with four or five levels of saliency per modality, we fit the behavioral response rates by fitting a criterion per modality and a d-prime for each saliency, which is described by a psychophysical function (three-parameter hyperbolic function). The d-prime at each saliency level follows from:

2 $${d}_{i}={d}_{{{{{\rm{max}}}}}}\ast{x}_{i}^{n}/\left({x}_{i}^{n}+{s}_{50}^{n}\right)$$

where *d*_max_ is the asymptotic d-prime, *s*_50_ is the stimulus strength at 50% of the asymptotic value, *n* is the slope of the psychometric function and *x*_*i*_ is the amount of change. This consisted of fitting a total of eight free parameters: *d*_max_, *n*, *s*_50_, and *c* for each modality. We refer the reader to Sridharan et al. for a detailed description of how the d-prime and criterion subsequently relate to response rates^40^. Single session fits where visual threshold was below 1° or above 45° were excluded (average threshold ±6°, *n* = 3/179 sessions excluded).

Catch trials during the tasks (in all cohorts) served to measure baseline lick responses. As there were no stimulus changes during the inter-trial interval (visual and auditory stimuli continued to be presented throughout the session, similarly to catch trials), we used long inter-trial intervals to insert additional artificial catch trials during offline analysis to improve the statistical balance across trial type conditions. We controlled for the temporal expectation of stimulus change and inserted additional catch trials only at time points conforming to the original inter-trial interval statistics. These additional catch trials included both false alarms (i.e., spontaneous licks during inter-trial intervals) and correct rejections (no spontaneous licks). We further constrained the timing of these false alarms such that the distribution of their response latencies matched those of hit trials. For sessions with high false alarm rates, FA trials were subsampled to match the distribution of hit trials. Note that these additional catch trials only served for analysis purposes and not for measuring behavioral performance.

After analyzing the effects of early and late V1 silencing on audiovisual change detection on the full dataset, we focused on a subsequent analysis of the relationship between reaction time and the effect of late silencing (Fig. 5i, j, Supplementary Fig. 6d–f). Here, we focused on sessions in which V1 early silencing was effective (minimum 50% reduction in d-prime on maximal visual change, 59/81 sessions; results were robust to variations of this criterion, 25% reduction, *r* = 0.199, *p* = 0.049; 75% reduction, *r* = 0.511, *p* = 0.001). This threshold was implemented to test if late silencing was effective specifically within those sessions in which the optogenetic manipulation demonstrably impaired visual detection (thus exploiting an internal control).

#### Behavioral analysis—Task B

Behavioral data in task B was fit with a multinomial logistic regression^90^. The probabilities of right choice (*p*_*right*_), left choice (*p*_*left*_), and no choice (*p*_*no-go*_) were set by:

3 $${{{{{\rm{log }}}}}}\left(\frac{{p}_{{right}}}{{p}_{{no}-{go}}}\right)={b}_{{right}}+{{sL}}_{{right}} * {{cL}}^{n}+{{sR}}_{{right}} * {{cR}}^{n}$$

4 $${{{{{\rm{log }}}}}}\left(\frac{{p}_{{left}}}{{p}_{{no}-{go}}}\right)={b}_{{left}}+{{sL}}_{{left}} * {{cL}}^{n}+{{sR}}_{{left}} * {{cR}}^{n}$$

where *b* is a bias parameter, *sL* and *sR* are the sensitivity to stimulus evidence to the left and right side respectively, *cL* and *cR* are the stimulus intensity to the left and right side respectively (contrast for vision, deflection angle for somatosensation), *n* is an exponent parameter between ranging between 0 and 1 to allow for saturation.

The model was fit to individual mice, with all sessions pooled together. However, per mouse, visual behavior (visual-only trials) and tactile behavior (tactile-only trials) were fit separately. The model was fit using Matlab’s *mnrfit* and maximum likelihood estimation. To quantify behavioral performance, we computed d-prime (*d*′) as:

5 $$d^{\prime}=\Phi^{\prime}( \% {Correct}\,{response}\,{to}\,{side}\,i){{\mbox{--}}}\Phi^{\prime}\left( \% {False}\,{alarm}\,{to}\,{side}\,i\right)$$

where *Φ*′ is the normal inverse cumulative distribution function.

#### Electrophysiological data processing

To visualize the effect of photostimulation on spiking activity at a single electrode channel the raw signal was high-pass filtered (500 Hz, 4th order Butterworth filter). To compute firing rates, spikes (following spike detection and sorting) were binned in 10 ms bins and convolved with a causal Half-Gaussian window with 50 ms standard deviation, unless stated otherwise. Wherever firing rate was z-scored, the mean was subtracted and divided by the standard deviation of the baseline period (−1 to −0.2 s before stimulus). For Fig. 5d the firing rate was only normalized to the baseline to quantify the relative reduction in firing rate by optogenetic inhibition. For Fig. 7g, h the standard deviation of the convolutional window was reduced to 10 ms to enhance temporal resolution. For computing noise correlations the standard deviation of the convolutional window was increased to 100 ms to increase noise correlation estimates. For Fig. 6c and Supplementary Fig. 8g, h, neurons with an average z-scored firing rate that exceeded 2 standard deviations at any point during the stimulus epoch of any visual trial condition were considered responsive and included. If neuronal activity was sampled in less than 3 trials in the relevant conditions they were excluded. Neurons in sessions lacking any of the compared conditions were excluded.

#### Encoding model of single-neuron firing rates

To quantify single neuron encoding of different task variables, we constructed a kernel-based Poisson regression model. This encoding model allowed us to model, for single neurons, the time-dependent effects of all measured variables related to the task and the animal’s behavior simultaneously on single-trial neuronal activity^42,43^. This approach is particularly useful to disentangle the unique contribution of experimenter-controlled task events and self-timed behavioral events to variability in firing rates across the neuronal population.

*Construction*—For each neuron, we constructed a design matrix based on five sets of variables; visual, auditory, hit, movement, and arousal variables. Binary variables (all except pupil size) were modeled with a series of temporal basis functions (raised cosines) that spanned the relevant epoch of influence. The number and temporal distribution of these basis functions were selected to maximize the cross-validated explained variance (see below). For the sensory predictors, we used two kernels with 100 ms standard deviation that spanned the first 200 ms post-stimulus to capture the early spiking activity and ten kernels with 200 ms standard deviation that spanned from 0 to 2000 ms post-stimulus to capture the late, sustained response. We found that making a separate predictor set per combination of orientation × amount of change produced the highest quality fit as it simultaneously took into account the selectivity of neurons for orientation and saliency. This therefore resulted in (2 + 10 basis functions) × 2 (modalities) × 2 (levels of change) × 2 (grouped post-change features) = 96 predictors. For hit variables, we used ten temporal basis functions with 200 ms standard deviation that spanned from 0 to 2000 ms relative to stimulus change in hit trials (visual hit, audio hit) and ten predictors that spanned −500 ms to +1500 ms relative to reward (20 predictors for hit). For movement variables, we used three basis functions that spanned −200 to +400 ms relative to each lick, split by side (six predictors). To capture arousal effects, the z-scored pupil area was included in the predictor set: with original timing and two temporal offsets (−800 ms and −400 ms) to account for the delayed relationship of brain state to pupil size (e.g., ref. ^91^; this equals three predictors). We included one whole-trial variable that scaled with the within-session trial number. This full model summed up to 126 predictors. We compared the performance of this model to a null model, with one predictor (a random variable). For convenience, all predictors were normalized to their maximum values before being fed into the model.

*Fitting*—We fitted the encoding model to each neuron’s activity individually, using the *glmnet* package in Matlab^92^ (2015 version) with elastic-net regularization and a Poisson link function, which involves setting three hyperparameters. First, we chose elastic net mixing parameter *α* = 0.95 to allow for a small number of uncorrelated informative predictors to be favored. Second, model performance was trained and tested on separate data with fivefold cross-validation. Third, to maximally punish weights without losing model fit quality, regularization parameter lambda was maximized while keeping the cross-validated error within one standard error of the minimum (lambda_1se in *glmnet*). Because very sparsely firing neurons produced fitting difficulties, only neurons with a session-average firing rate >0.5 Hz were included.

*Evaluation*—We quantified the model performance by assessing the fivefold cross-validated Explained Variance (EV) by the predicted firing rate based on the random or full model, or a subset of predictors from the full model. EV was calculated as:

6 $${{{{{\rm{EV}}}}}}=1-\frac{{{{{{\rm{var}}}}}}(Y-\hat{Y})}{{{{{{\rm{var}}}}}}(Y)}$$

where *Y* is the original firing rate and *Ŷ* the estimated firing rate. Explained Variance was computed in two ways. First, we computed EV overall concatenated firing rate bins (overall single trials; −0.5 to 2.5 s relative to stimulus change). Second, we computed EV on the concatenated firing rate bins of the average firing rate for five trial-type × choice conditions that captured most trial counts^25,43^ (85% of all trials). To compute EV over time we computed the explained variance overall concatenated time bins at a specific moment relative to stimulus onset.

#### Decoding single-neuron activity

To identify which variables could be decoded from single neurons we used ROC analysis^45^ and identified how well an external observer could discriminate variables from the firing rate at single time points. In contrast to the GLM encoding approach, this analysis is more suited to assess at which timepoint downstream neurons can discriminate task-relevant variables (e.g., by integrating spike rate over a small time window), in view of its superior temporal resolution due to the absence of kernel fitting. We computed the area under the ROC curve (AUC) for the firing rate distributions between two selections of trials. Each class had to have at least ten trials. AUC values are in the range of 0 to 1 and were rectified to their corresponding values in the range between 0.5 and 1. We investigated three types of coding and for each of these we analyzed threshold change and maximum change trials separately:

*Visual Orientation*: We grouped the pairs of post-change orientations that were close to each other (e.g., A and B oriented at 90° and 97°, see above) and thus compared the firing rate distributions of A&B versus C&D for threshold and maximal change trials separately.

*Occurrence of visual change*: We tested whether single neuron firing rates discriminated between visual and catch trials.

*Hit/miss*: To identify significant coding of the detection of a visual stimulus we compared firing rate distributions within visual trials for hits and misses.

To determine the significance of AUC values at each time bin and for each comparison, we performed a permutation test by shuffling the class labels across trials 1000 times. If the unshuffled AUC value exceeded 99% of the shuffled distribution (*P* < 0.01) this was deemed significant. This yielded an AUC value for each neuron for each time bin for each type of coding and each of these values its significance by the permutation test. For the Venn diagram in Fig. 4A, neurons were classified as coding if their AUC score was significant for at least three consecutive time bins (=75 ms, permutation test) during the 0–1000 ms window after a stimulus change.

To compare coding dynamics across cohorts, we normalized the fraction of significantly coding neurons by subtracting the baseline fraction (average over −0.5 to 0 s) and dividing by the maximum. Each condition was only normalized to maximum if the fraction of significantly coding neurons increased at least 10% over baseline.

To determine the onset of significant coding we tested when the fraction of coding neurons increased significantly above a multiple of standard deviations of the coding fraction during baseline. We report results at a threshold of 2 standard deviations (Z score > 2), but the results were robust to variations in threshold (e.g., 1 or 3 standard deviations). To estimate the reliability of the onset of coding and the relationship of hit/miss coding to reaction time, we bootstrapped by resampling from the total neuronal population (*n* = 1000 bootstraps). To investigate the relationship between the onset of hit/miss coding and reaction time we used a linear regression, which revealed a systematic relationship between the timing of hit/miss coding and reaction time (Fig. 4e, f). To estimate by how much hit/miss coding preceded reaction time we used two measures. First, we fixed the slope of the regression fit at 1 and found an offset of 278 ms. This was similar across variations of threshold (*Z* > 1: 288 ms, *Z* > 3: 250 ms). Second, for each bootstrap, we computed the onset of hit/miss- coding according to the fit parameters for the average reaction time, which was on average 266 ms before the reaction time.

Identifying the onset of coding by using the fraction of neurons coding above baseline was not suitable for individual sessions with low numbers of neurons (e.g., *n* = 10 neurons). Therefore, to estimate the onset of hit/miss coding per session we performed the same AUC analysis, but now on the averaged firing rate across neurons for each session with at least ten neurons. Similarly, per time bin the significance of hit/miss coding was tested against a shuffled distribution of trial labels (*n* = 1000 shuffles). The onset was taken as the first significant time bin after stimulus onset.

For laminar depth localization of coding dynamics, neurons were binned according to their recorded depth in 50 μm bins spanning from 0 to 1150 μm below the dura. The fraction of neurons coding for each variable at this depth was computed for each time point (25 ms temporal bins). This heatmap was convolved for display purposes with a two-dimensional Gaussian (standard deviation of 1.3 bins—temporal and spatial). For statistical comparison across laminar zones, the fraction of coding neurons was computed for each session (if at least ten neurons were recorded at this depth to estimate coding fraction reliably) in supragranular, granular, or infragranular layers (granular layer: 400–550 μm from dura). As sensory and hit/miss-coding was present in different temporal epochs these were included for statistical comparison (Orientation 0–1000 ms, Visual occurrence: 0–200 ms, Hit/miss: 200–1000 ms, relative to stimulus change).

#### Population coding analysis

To decode visual stimulus orientation, we departed from the four orientations and grouped the two pairs of orientations close to each other to obtain a two-class classification problem (AB vs CD, see above). Decoding was performed on recordings that contained at least 15 neurons and in which at least 20 trials per orientation pair were available. We equalized the number of neurons across sessions by randomly drawing ten neurons from all sessions with more than ten units. Spikes were binned using a sliding window of 200 ms with 50 ms increments, excluding time bins that contained both pre and post-stimulus spikes. Decoding was performed using a random forest classifier with 200 trees, as implemented in Scikit-learn^93^ (version 0.23.0), and we employed a 5 × 5 cross-validation routine with stratified folds (cf.^94^). The average accuracy obtained in the cross-validation routine was corrected by subtracting the average accuracy on 50 surrogate datasets in which the orientation labels were permuted across trials to obtain the improvement in decoding accuracy beyond chance level.

#### Noise correlations

To investigate correlated activity across the population we computed pairwise correlations on the binned spike counts (10 ms bins, time range: −1000 to +1500 ms relative to stimulus change) after subtracting the average stimulus-driven response. First, for each neuron the trial-mean firing rate over time was subtracted for all subsets of trials of interest (per orientation). Next, the Pearson’s correlation coefficient was computed between the residual rates for each simultaneously recorded neuronal pair for each time bin. Neuronal pairs for a given condition were included if they were sampled in more than 10 trials. We also computed pairwise correlations aligned to lick onset and thus subtracted mean activity related to lick-related modulation of firing rate. We note, however, that the term ‘noise correlations’ is conventionally reserved for the correlations on residual rates after subtracting the mean stimulus-evoked activity (rather than movement-evoked activity). Note also that drop in noise correlations was not a direct result of overall increased firing rates as we observed no such reduction of noise correlations during early sensory-evoked activity (0–200 ms). Neurons were only included if their session average firing rate was above 1 Hz.

#### Statistics

Unless specified otherwise all statistics were performed using linear mixed models (LMMs) or generalized linear mixed models (GLMMs) in MatLab (MathWorks, Natick, MA). (G)LMMs take into account the hierarchical nature of our data (neurons and trials sampled from the same mice)^95^. (G)LMMs describe the relationships between a response variable and multiple explanatory variables, and comprise two types of explanatory terms. Fixed effects are the variables of interest, while random effects, also commonly referred to as grouping variables, specify and account for the nesting group (mouse ID in our case). For all analysis involving hierarchical data, LMMs were constructed with mouse ID as a random effect (intercept only). Importantly, mouse ID was not included as a random effect for analyses with cohort as fixed effect, as variability between mice was key to those results. As firing rates are generally not normally distributed, for analyses of firing rate responses (non z-scored) we used Generalized Linear Mixed Models with a Poisson distribution. Statistical tests were performed on the fixed effect using ANOVAs on the (G)LMMs. To estimate the denominator degrees of freedom for *F*-tests, the Satterthwaite approximation was used for LMMs and the residual degrees of freedom for GLMMs. Linear hypothesis tests were performed in the case of post hoc comparisons using the relevant contrasts. A description of each test, sample sizes, test statistics, and *p* values are provided in Supplementary Table 1. Results with a *p* value lower than 0.05 were considered significant.

To verify whether our cohort-specific results were likely to be a consequence of a difference in group size, we subsampled MST neurons and sessions to match UST sample size for two key results (Figs. 4d, 5h) and found that our results were maintained (1000 resamples, see Supplementary Table 1).

### Reporting summary

Further information on research design is available in the Nature Research Reporting Summary linked to this article.

## Supplementary information

Supplementary Information

Reporting Summary

## Data Availability

All behavioral and neural data related to this study are openly available at https://gitlab.com/csnlab/olcese-lab/modid-project/2nd-bump and have been deposited on Zenodo (10.5281/zenodo.6451263). Source data are provided in this paper.

## Source data

Source Data

## Code availability

All code related to this study is openly available at https://gitlab.com/csnlab/olcese-lab/modid-project/2nd-bump. The version of the code used in this study is available on Zenodo (10.5281/zenodo.6451263).
