# Author Correction: Multisensory task demands temporally extend the causal requirement for visual cortex in perception

**DOI:** 10.1038/s41467-022-31518-7

**Published:** 2022-07-14

**Authors:** Matthijs N. Oude Lohuis, Jean L. Pie, Pietro Marchesi, Jorrit S. Montijn, Christiaan P. J. de Kock, Cyriel M. A. Pennartz, Umberto Olcese

**Affiliations:** 1 Cognitive and Systems Neuroscience, Swammerdam Institute for Life Sciences, University of Amsterdam, Amsterdam, The Netherlands; 2 Research Priority Area Brain and Cognition, University of Amsterdam, Amsterdam, The Netherlands; 3 Department of Integrative Neurophysiology, Center for Neurogenomics and Cognitive Research, VU Amsterdam, Amsterdam, The Netherlands; 4 Cortical Structure and Function, Netherlands Institute for Neuroscience, Amsterdam, The Netherlands

**Keywords:** Sensory processing, Striate cortex, Perception

Correction to: *Nature Communications* 10.1038/s41467-022-30600-4, published online 23 May 2022.

The original version of this Article incorrectly included the 6th author Cyriel M. A. Pennartz as a corresponding author. The correct version lists only 7th author Umberto Olcese as corresponding author. In addition, the original version of the Article omitted to include a statement to indicate that Cyriel M. A. Pennartz and Umberto Olcese jointly supervised the work. This has been corrected in both the PDF and HTML versions of the Article.

