# Supplementary material for: Multisensory task demands temporally extend the causal requirement for visual cortex in perception
Source: Nat Commun. 2022 May 23;13:2864. doi: 10.1038/s41467-022-30600-4 (PMC9126973; doi:10.1038/s41467-022-30600-4)
Supplement: Supplementary file 1 — Supplementary Information [file 41467_2022_30600_MOESM1_ESM.pdf]

Multisensory task demands temporally extend the causal requirement for visual cortex in perception

AUTHOR LIST:

Matthijs N. Oude Lohuis, Jean L. Pie, Pietro Marchesi, Jorrit S. Montijn, Christiaan P.J. de Kock, Cyriel M. A. Pennartz, Umberto Olcese

Supplementary Table 1 Statistical tests

Figure	Goal	Test	Sample sizes	Test statistic	p-value (significant in bold)
1f	Visual d-prime UST vs MST	Linear Mixed Model ANOVA (fixed effect: cohort)	12 UST sessions, 139 MST sessions	F(1,29) = 1.60, p=0.22	p=0.22
1f	Auditory vs visual d-prime in MST	Linear Mixed Model ANOVA (fixed effect: modality, random effect: mouse ID)	139 MST sessions	F(1,261) = 36.26	p=5.84e-9
1g	Visual threshold UST vs MST	Linear Mixed Model ANOVA (fixed effect: cohort)	12 UST sessions, 139 MST sessions	F(1,31) = 0.45	p=0.51
Not shown	Visual sensitivity UST vs MST	Linear Mixed Model ANOVA (fixed effect: cohort)	12 UST sessions, 139 MST sessions	F(1,31)=3.74	p=0.06
1h	Linear dependence between visual saliency and RT (UST)	Linear Mixed Model ANOVA (fixed effect: saliency, random effect: mouse ID)	493 trials	F(1,482) = 5.81	p=0.02
	Linear dependence between visual saliency and RT (MST)		3424 trials	F(1,3371) = 144.67	p=1.17e-32
	Linear dependence between auditory saliency and RT (MST)		4276 trials	F(1,4219) = 16.52	p=4.91e-05
1h	Auditory vs visual RT (MST)	Linear Mixed Model ANOVA (fixed effect: modality, controlling for saliency, random effect: mouse ID)	3424 visual trials, 4276 auditory trials	F(1,7599) = 706.89	p=5.28e-149
1h	Visual RT for UST vs MST	Linear Mixed Model ANOVA (fixed effect: cohort, controlling for saliency)	3917 trials (493 UST + 3424 MST)<br>Subthreshold: 71 UST trials, 483 MST trials<br>Threshold: 115 UST trials, 748 MST trials<br>Suprathreshold: 130 UST trials, 933 MST trials<br>Max: 168 UST trials, 1217 MST trials	F(1,3865) = 60.17	p=1.11e-14
2f	Maximal z-cored response	Linear Mixed Model ANOVA	Supragranular - 91 neurons	F(1,194) = 4.60	p=0.03

	early vs late phase per laminar zone	(fixed effect: temporal window, random effect: mouse ID)	Granular - 88 neurons	F(1,171) = 0.00	p=0.96
			Infragranular: 582 neurons	F(1,1284) = 23.32	p=1.53e-06
2f	Hit/miss modulation different across laminar zone	Linear Mixed Model ANOVA (fixed effect: laminar zone, random effect: mouse ID)	91+88+592=771 neurons	F(2,771) =4	p=0.019
				F(1,784)=12.97<br>F(1,784)=6.50<br>F(1,784)=0.58	G vs SG: p=0.0033<br>G vs IG: p=0.01<br>IG vs SG: p=0.45
4c	Fraction of neurons coding (significant AUC) different across layers	Linear Mixed Model ANOVA (fixed effect: laminar zone, random effect: mouse ID)<br><br>Posthoc comparison (Linear hypothesis test on coefficients)<br><br>(Only significant reported, rest p>0.05)	Visual orientation; no significant layer differences.		p>0.05
			Visual change occurrence; 4 sessions with enough supragranular neurons, 14 sessions with enough infragranular neurons,	F(1,16)=7.21	p=0.016
			Visual hit/miss threshold; 3 sessions with enough granular neurons, 15 sessions with enough infragranular neurons	F(1,15)=5.21	p=0.037
			Visual hit/miss maximal; 3 sessions with enough granular neurons, 15 sessions with enough infragranular neurons	F(1,15)=4.96	p=0.042
4d	Earliest time point of increase in coding fraction	Fraction significant neurons exceeds 2 std above baseline (-1000 to 0 ms). This corresponds to a one-sided t-test with p < .02275. Very similar results were obtained with a threshold of 1 or 3 std above baseline.	NE: 116 neurons<br><br>UST: 128 neurons<br><br>MST: 306 neurons	Z>2	p<0.02275
4d	For each variable, the difference in latency to coding between cohorts	Bootstrap test (n=1000 resamples). If the difference between bootstrap distribution exceeded the 97.5 percentile this was deemed significant. This corresponds to a two-sided p-value of 0.05.	NE: 116 neurons<br><br>UST: 128 neurons<br><br>MST: 306 neurons		Hit/miss coding latency threshold changes between UST and MST: P=0.012 Rest p>0.05.
	Subsample control (4d)	Bootstrap test on n=1000 resamples with the same number of neurons between UST and MST.	UST: 128 neurons<br><br>MST: 128/306 neurons		Hit/miss coding latency threshold changes between UST and MST p<0.05. Rest p>0.05.
4e	Linear dependence earliest hit/miss coding of population activity and RT (on individual sessions)	Linear model ANOVA	26 sessions	F(1,24) = 5.15	p=0.03
4f	Linear dependence earliest increase in visual hit/miss coding and RT (on bootstrapped condition averages)	Linear model ANOVA	4 conditions (UST and MST, 2 saliencies each)<br>RT boostrapped from n=1269 Vthr UST trials, n=1292 Vmax UST trials, n=960 Vthr MST trials, n=1051 Vmax MST trials<br>Coding onset boostrapped from n= 128 UST V1 neurons, n=306 MST neurons.	F(1,2) = 102.33	p=0.0096
			Bootstrap results: (Mean and 95% CI)		Slope: 1.58 (0.27-2.52)<br>Offset: -569 ms (-985 to -10).
5g,5h	Effect of inactivation on discrimination performance (d-prime) comparing early or late	Linear Mixed Model ANOVA (fixed effect of inactivation, random effect: mouse ID) with Bonferroni-Holm correction for	threshold visual change, UST, Ctrl vs Early<br>18 sessions	F(1,32)=16.71	p=0.0032
			threshold visual change, UST, Ctrl vs Late<br>18 sessions	F(1,32)=0.29	p=1

	inactivation versus control trials for different saliencies, modalities, and cohorts	multiple comparisons (4 comparisons)	threshold visual change, MST, Ctrl vs Early<br>34 sessions	F(1,59)=35.32	p=0.000002
			threshold visual change, MST, Ctrl vs Late<br>34 sessions	F(1,54)=13.90	p=0.00553
			maximum visual change, UST, Ctrl vs Early<br>18 sessions	F(1,32)=14.80	p=0.0064
			maximum visual change, UST, Ctrl vs Late<br>18 sessions	F(1,30)=1.19	p=0.85
			maximum visual change, MST, Ctrl vs Early<br>34 sessions	F(1,58)=32.56	p=0.000005
			maximum visual change, MST, Ctrl vs Late<br>34 sessions	F(1,53)=13.48	p=0.0067
			auditory change, MST, all comparisons<br>34 sessions	All F < 3	All p>0.1
5g,5h (not shown)	Subsample control of 5g,h	Same as above but for n=1000 resamples of 18/34 MST sessions to match # of UST session	threshold visual change, MST, Ctrl vs Late<br>18/34 MST sessions		P<0.05 for 82% of resamples
			maximal visual change, MST, Ctrl vs Late<br>18/34 MST sessions		P<0.05 for 86% of resamples
5i	Linear dependence median RT and percentage reduction d-prime	Linear Mixed Model ANOVA (fixed effect of reaction time, random effect: mouse ID)	Early silencing: 40 conditions (21 Thr and 19 Max)	F(1,33)=1.71	p=0.20
5j	Linear dependence median RT and percentage reduction d-prime	Linear Mixed Model ANOVA (fixed effect of reaction time, random effect: mouse ID)	Late silencing: 45 conditions (22 Thr and 23 Max)	F(1,15)=10.04	P=0.006
5j (not shown)	Linear dependence median RT and percentage reduction d-prime (controlling for visual dprime on control trials)	Linear Mixed Model ANOVA (fixed effect of reaction time and visual dprime; random effect: mouse ID)	Late silencing: 45 conditions (22 Thr and 23 Max)	RT: F(1,20)=11.77,<br>Dprime: F(1,42)=1.93	p=0.003<br><br>p=0.172
6d	Effect of inactivation on D-prime. Comparing Early or Late inactivation versus control trials for different saliencies, modalities, sides, cohorts.	Linear Mixed Model ANOVA (fixed effect: inactivation, random effect: mouse ID) with Bonferroni-Holm correction for multiple comparisons (4 comparisons)	Visual contralateral threshold detection, UST, Ctrl vs Early<br>7 sessions	F(1,14)=24.57	P=6.3179e-04
			Visual contralateral threshold detection, UST, Ctrl vs Late<br>7 sessions	F(1,14)=2.15	P=0.1644
			Visual contralateral threshold detection, MST, Ctrl vs Early<br>6 sessions	F(1,12)=17.93	P= 0.0023
			Visual contralateral threshold detection, MST, Ctrl vs Late<br>7 sessions	F(1,14)=45.14	P=3.6441e-05
		Linear Mixed Model ANOVA (fixed effect: inactivation, random effect: mouse ID) with Bonferroni-Holm correction for multiple comparisons (4 comparisons)	Visual contralateral maximum detection, UST, Ctrl vs Early<br>7 sessions	F(1,14)=0.16	P=0.9656
			Visual contralateral maximum detection, UST, Ctrl vs Late<br>7 sessions	F(1, 9)=0.53	P=0.9656
			Visual contralateral maximum detection, MST, Ctrl vs Early<br>4 sessions	F(1, 8)=3.73	P=0.2636
			Visual contralateral maximum detection, MST, Ctrl vs Late<br>7 sessions	F(1,13)=6.38	P=0.1023
		Linear Mixed Model ANOVA (fixed effect: inactivation, random effect: mouse ID) with	Visual ipsilateral threshold detection, UST, Ctrl vs Early<br>7 sessions	F(1, 7)=0.04,	P=1

		Bonferroni-Holm correction for multiple comparisons (4 comparisons)	Visual ipsilateral threshold detection, UST, Ctrl vs Late<br>7 sessions	F(1,16)=0.80	P=1
			Visual ipsilateral threshold detection, MST, Ctrl vs Early<br>2 sessions	F(1,10)=0.02	P=1
			Visual ipsilateral threshold detection, MST, Ctrl vs Late<br>7 sessions	F(1,10)=0.38	P=1
6e	Linear dependence between percentage reduction d-prime and reaction time	Linear Mixed Model ANOVA (fixed effect: median RT, random effect: mouse ID)	30 conditions (7 UST thr, 7 UST high, 9 MST low, 7 MST high)	F(1,26)=9.78	p=0.004<br>r^2= 0.7056
7b	Pre-stim (-500 to 0 ms) vs post-stim (0 to +500 ms) decoding improvement over chance	Linear Mixed Model ANOVA (fixed effect: time window, random effect: mouse ID)	11 sessions combined across cohorts	F(1,17)=44.76	p=4.118e-06
7c	Significant decrease in noise correlations versus baseline for visual trials split by choice	Linear Mixed Model ANOVA (fixed effect of choice; random effect: mouse ID)	NE – Miss trials, 2904 pairs	F(1,5805)=14.67	P<1e-4
			NE – Hit trials, 1476 pairs	F(1,2950)=0.33	p=0.56
			UST – Miss trials, 1930 pairs	F(1,3856)=0.02	p=0.88
			UST – Hit trials, 1930 pairs	F(1,3856)=82.44	p<1e-19
			MST – Miss trials, 13692 pairs	F(1,27467)=3.31	p=0.069
			MST – Hit trials, 13972 pairs	F(1,28188)=142.96	P<1e-33
7d	Difference in visual hit reaction time between cohorts (median RT of session)	Two-sided Wilcoxon rank-sum test	11 UST sessions vs 44 MST sessions		p=0.0041
7e	Earliest time point of decorrelation	Earliest time point that noise correlations drop below baseline minus two standard deviations values. This corresponds to a one-sided t-test with p<0.05. Similar results were obtained with more or less strict thresholds.	From 59 sessions:<br>UST: 4730 neuron pairs MST: 17826 neuron pairs		Z<-2
7f	Linear dependence reaction time and earliest time point of decorrelation	Pearson correlation	6 condition averages (fast, mid and slow tertiles for UST and MST each)		r=0.960,<br>p=0.002
7g	Difference in z-scored activity between hit-miss during 100-200 ms window	Linear Mixed Model ANOVA (fixed effect: hit/miss, random effect: mouse ID)	UST - Thr - 78 neurons	F(1,156)=10.16	P=0.002
			UST - Max - 78 neurons	F(1,152)=5.66	P=0.019
7h	Difference in z-scored activity between hit-miss during 100-200 ms window	Linear Mixed Model ANOVA (fixed effect: hit/miss, random effect: mouse ID)	MST - Thr - 134 neurons	F(1,268)=13.19	P=0.001
			MST - Max - 120 neurons	F(1,254)=0.59	P=0.445
7i	Difference in noise correlation for each time bin	Two-sided bootstrapped confidence interval test	230 UST and MST neurons, 1564 neuron pairs, 1000 bootstraps		Black lines in Figure 5i, indicate P<0.05
	Supplementary figures:				
S2f	Linear dependence between auditory d-prime	Linear Mixed Model ANOVA (fixed effect: RT, controlling for	139 MST sessions, 4 saliencies each	F(1,298) = 10.43	p=0.00138

	and RT	saliency; random effect: mouse ID)			
S2g	Linear dependence between visual d-prime and RT	Linear Mixed Model ANOVA (fixed effect: RT, controlling for saliency; random effect: mouse ID)	151 UST and MST sessions, 4 saliencies each	F(1,268) = 11.36	p=0.00086
S3b	Difference in trial-averaged z-scored firing rate in 0 to 200ms post-stimulus window between threshold and maximal visual change conditions per cohort	Linear Mixed Model ANOVA (fixed effect: saliency, random effect: mouse ID)	163 NE neurons	F(1,326) = 7.27	P=0.0079
			128 UST neurons	F(1,256) = 20.43	p=9.46e-06,
			525 MST neurons	F(1,1568) = 35.90	p=2.56e-09
S3i	Difference in trial-averaged z-scored firing rate in -300 to 300ms window between lick and no-lick conditions	Linear Mixed Model ANOVA: Fixed effect of Licking: F(1,1966)=379.35, p=2.13e-77; Fixed effect of Cohort: F(2,1966)=6.08, p=0.002; Interaction effect Licking * Cohort: (2,1966)=10.59, p=2.658e-05;	163 NE neurons,<br>128 UST neurons<br>525 MST neurons;	Posthoc comparison: Linear hypothesis test on coefficients	p-value in figure, *p<0.05, **p<0.01, ***p<0.001
			Specific posthoc comparison interaction term Licking*Cohort for NE versus MST&UST.	F(1,1960)=19.71,	p=9.526e-06
S3j	Difference in trial-averaged z-scored firing rate in -300 to 300ms window between hit and incorrect conditions for trained UST and MST conditions	Linear Mixed Model ANOVA (fixed effect: Correct, random effect: Mouse ID):	Visual Incorrect vs Hits, n = 128 UST neurons	F(1,256) = 2.59	p=0.109
			Visual Incorrect vs Hits, n = 525 MST neurons	F(1,1371) = 140	P=2.54e-08
			Auditory Incorrect vs Hits, n = 525 MST neurons	F(1,1371) = 9.67	p=0.002
S4a	Difference in explained variance over single trials between cohorts over all trials using all predictors	Linear Mixed Model ANOVA (Fixed effect: cohort):<br><br>(F(2,516) = 4.71, p=0.01, ANOVA)<br><br>Posthoc comparison: Linear hypothesis test on coefficients	n=116 NE neurons, n=128 UST neurons, n=272 MST neurons, (only neurons >0.5 Hz)<br><br>NE vs UST	F(1,513)=0.81	p=0.36
			NE vs MST	F(1,513)=8.28	p=0.004
			UST vs MST	F(1,513)=3.62	p=0.058
S4b	Difference in explained variance over averaged trial types between cohorts over all trials using all predictors	Linear Mixed Model ANOVA (fixed effect: cohort):<br><br>(F(2,516) = 7.01, p=0.001, ANOVA)<br><br>Posthoc comparison: Linear hypothesis test on coefficients	(n same as Fig. S4a)<br><br>NE vs UST	F(1,513)=1.40	p=0.23
			NE vs MST	F(1,513)=12.55	p=0.0004
			UST vs MST	F(1,513)=5.06	p=0.025
S4c	Explained variance averaged over 0-200ms (early activity) between predictor sets within cohort	Linear Mixed Model ANOVA (fixed effect: cohort with posthoc comparison: Linear hypothesis test on coefficients)	n=116 NE neurons<br><br>n=128 UST neurons<br><br>n=272 MST neurons	Vision :<br><br>(MST vs NE):F(1,513)=16.60<br><br>(NE vs UST):F(1,513)=22.40<br><br>(MST vs UST):F(1,513)=2.09<br><br>Movement:<br><br>(MST vs NE):F(1,513)=23.16,<br><br>(NE vs UST):F(1,513)=6.28,<br><br>(MST vs UST):F(1,513)=3.93,	Vision :<br><br>(MST vs NE) p=0.000053<br><br>(NE vs UST) p=0.0000028<br><br>(MST vs UST) p=0.14888<br><br>Movement:<br><br>(MST vs NE) p=0.0000019<br><br>(NE vs UST) p=0.0125<br><br>(MST vs UST) p=0.0480

				Hit:<br><br>(UST vs NE):F(1,513)=39.48<br><br>(MST vs NE):F(1,513)=2.02<br><br>(MST vs UST):F(1,513)=23.06<br><br>Pupil:<br><br>(MST vs NE):F(1,513)=0.17<br><br>(NE vs UST):F(1,513)=3.05<br><br>(MST vs UST):F(1,513)=6.32	Hit:<br><br>(UST vs NE) p=0.0000000007<br><br>(MST vs NE) p=0.1562<br><br>(MST vs UST) p=0.000002<br><br>Pupil:<br><br>(MST vs NE) p=0.6800447193<br><br>(NE vs UST) p=0.0815248918<br><br>(MST vs UST) p=0.0122326371
S4d	Explained variance averaged over 200-1000ms (late activity) between predictor sets within cohort	Linear Mixed Model ANOVA (fixed effect: cohort with posthoc comparison: Linear hypothesis test on coefficients)	n=116 NE neurons<br><br>n=128 UST neurons<br><br>n=272 MST neurons	Vision :<br><br>(MST vs NE):F(1,513)=9.14<br><br>(NE vs UST):F(1,513)=12.27<br><br>(MST vs UST):F(1,513)=1.13<br><br>Movement:<br><br>(MST vs NE):F(1,513)=32.08<br><br>(NE vs UST):F(1,513)=7.45<br><br>(MST vs UST):F(1,513)=6.74<br><br>Hit:<br><br>(MST vs NE):F(1,513)=53.50<br><br>(NE vs UST):F(1,513)=41.98<br><br>(MST vs UST):F(1,513)=0.03<br><br>Pupil:<br><br>(MST vs NE):F(1,513)=0.15<br><br>(NE vs UST):F(1,513)=1.27<br><br>(MST vs UST):F(1,513)=0.91	Vision :<br><br>(MST vs NE) p=2.632963e-03<br><br>(NE vs UST) p=5.003239e-04<br><br>(MST vs UST) p=2.885020e-01<br><br>Movement:<br><br>(MST vs NE) p=2.470784e-08<br><br>(NE vs UST) p=6.573378e-03<br><br>(MST vs UST) p=9.705983e-03<br><br>Hit:<br><br>(MST vs NE) p=1.001216e-12<br><br>(NE vs UST): p=2.162020e-10<br><br>(MST vs UST): p=8.557330e-01<br><br>Pupil:<br><br>(MST vs NE): p=7.017013e-01<br><br>(NE vs UST) p=2.596394e-01<br><br>(MST vs UST) p=3.409608e-01
S4e	Onset latency of significant hit encoding different for UST and MST cohorts<br><br>Onset latency = time bin where EV exceeded baseline + 2 standard deviations	Linear Mixed Model ANOVA (fixed effect: modality)	n=128 UST neurons<br><br>n=272 MST neurons	F(1,337) = 1.54	p=0.21
S6b	Effect of inactivation on discrimination performance (d-prime)	Linear Mixed Model ANOVA (fixed effect of inactivation,	Auditory change, MST, all comparisons	All F < 6	All p>0.1

	comparing early or late inactivation versus control trials for auditory saliencies	random effect: mouse ID)	34 sessions		
S6e	Effect of inactivation on discrimination performance (d-prime) comparing early or late inactivation versus control trials for visual saliencies, for sessions with the fastest average RT (top 50%)	Linear Mixed Model ANOVA (fixed effect of inactivation, random effect: mouse ID)	n=18 sessions	Visual thr change MST, Ctrl vs Early: F(1,31)=29.15	p=0.000027
				Visual max change MST, Ctrl vs Early:: F(1,25)=46.00	p=0.000002
				Visual thr change MST, Ctrl vs Late: F(1,27)=2.18	p=0.606787
				Visual max change MST, Ctrl vs Late: F(1,28)=9.05	p=0.022190
S6h	Effect of inactivation on discrimination performance (d-prime) comparing early or late inactivation versus control trials for visual saliencies, for sessions with the slowest average RT (bottom 50%)	Linear Mixed Model ANOVA (fixed effect of inactivation, random effect: mouse ID)	n=17 sessions	Visual thr change MST, Ctrl vs Early: F(1,30)=11.76	p=0.007136
				Visual max change MST, Ctrl vs Early: F(1,30)=5.34	p=0.111827
				Visual thr change MST, Ctrl vs Late: F(1,31)=16.31	p=0.001313
				Visual max change MST, Ctrl vs Late: F(1,29)=6.98	p=0.0047151
S6k	Linear dependence median RT and percentage reduction in criterion	Linear Mixed Model ANOVA (fixed effect of RT, random effect: mouse ID)	87 conditions (45 Thr and 42 Max)	F(1,39)=1.55	p=0.22
S6fk (not shown)	Linear dependence median RT and FA visual lick spout	Linear Mixed Model ANOVA (fixed effect of RT, random effect: mouse ID)	94 conditions (47 Thr and 47 Max)	F(1,46)=0.05	p=0.82
S6l	Late silencing delays reaction times: difference in reaction time between control and late silencing visual hits	Generalized Linear Mixed Model ANOVA (fixed effect of inactivation, random effect: mouse ID)	maximal changes: Control hits (n=795 trials) and late silencing hits (n=388 trials)	F(1,1181)=3.12	p=0.08
			threshold changes: Control hits (n=509 trials) and late silencing hits (n=252 trials)	F(1,759)=0.28	p=0.59
S7b-c	D-prime visual and audio change, UST and MST, Ctrl vs Early and Ctrl vs Late (S1 inactivation)	Linear Mixed Model ANOVA (fixed effect of inactivation, random effect: mouse ID) with Bonferroni-Holm correction for multiple comparisons (4 comparisons)	13 UST sessions, 16 MST sessions	All F < 6	Non-significant, all comparisons p>0.05

S8a	D- prime maximum, visual UST vs MST	Linear Mixed Model ANOVA (fixed effect: Cohort, controlling for side).	UST: 4 mice x 7 sessions x 2 sides<br><br>MST: 4 mice x 15 sessions x 2 sides	F(1,44)=3.1822	P=0.0814
S8b	Visual detection threshold, UST vs MST	Linear Mixed Model ANOVA (fixed effect: Cohort, controlling for side)	MST: 4 mice x 2 sides<br><br>UST: 4 mice x 2 sides	F(1,16)=0.3675	p=0.5529
S8c	Visual RT, UST vs MST	Generalized Linear Mixed Model ANOVA (fixed effect: cohort, controlling for saliency and side) with Bonferroni-Holm correction for multiple comparisons (2 comparisons)	1395 trials	F(1,1391)= 54.075	p=3.2832e-13
	Visual RT (UST), Thr vs Max	Generalized Linear Mixed Model ANOVA (fixed effects: Saliency, controlling for Side, random effects: session, mouse) with Bonferroni-Holm correction for multiple comparisons (2 comparisons)	617 trials	F(1,614)= 129.85	P=4.0354e-27
	Visual RT (MST), Thr vs Max		778 trials	F(1,775)= 56.919	P=2.5486e-13
	Tactile RT (MST), Thr vs Max	Generalized Linear Mixed Model ANOVA (fixed effects: Saliency, controlling for Side, random effects: session, mouse)	625 trials	F(1,622) = 5.403	P= 0.020424
S8d	D-prime tactile contralateral, threshold, MST, Ctrl vs Early	Linear Mixed Model ANOVA (fixed effect: inactivation, random effect: mouse ID) with Bonferroni-Holm correction for multiple comparisons (2 comparisons)	6 sessions	F(1,12)=3.78	p=0.1513
	D-prime tactile contralateral, threshold, MST, Ctrl vs Late		9 sessions	F(1,14)=0.12	p=0.7339
S8e	Effect of inactivation on the percentage of right-sided licks for condition: Thr Contralateral Visual	Linear Mixed Model ANOVA (fixed effect: inactivation, random effect: mouse ID) with Bonferroni-Holm correction for multiple comparisons (4 comparisons)	UST, Early vs Ctrl, 7 sessions	F(1,10.7)=39.08	P = 2.0943e-04
			UST, Late vs Ctrl, 7 sessions	F(1,10.7)=7.805	P = 0.0178
			MST, Early vs Ctrl, 7 sessions	F(1,12)=32.456	P = 2.0943e-04
			MST, Late vs Ctrl, 6 sessions	F(1,18)=38.242	P = 3.0993e-05
S9a	Significant decrease in noise correlations versus baseline for audio trials split by choice	Linear Mixed Model ANOVA (fixed effect of choice, random effect: mouse ID)	NE – Miss trials, 2904 pairs	F(1,5805)=1.96	P=0.16
			NE – Hit trials, 2106 pairs	F(1,1748)=3.83	p=0.054
			MST – Miss trials, 13656 pairs	F(1,27395)=22.61	p=1.99e-6
			MST – Hit trials, 14462 pairs	F(1,28847)=99.90	P=1.7e-23
S9b	Linear dependence reaction time and earliest time point of decorrelation relative to first lick	Pearson correlation	6 condition averages (fast, mid and slow tertiles for UST and MST each)	r=0.738	p=0.094

SUPPLEMENTARY FIGURES:

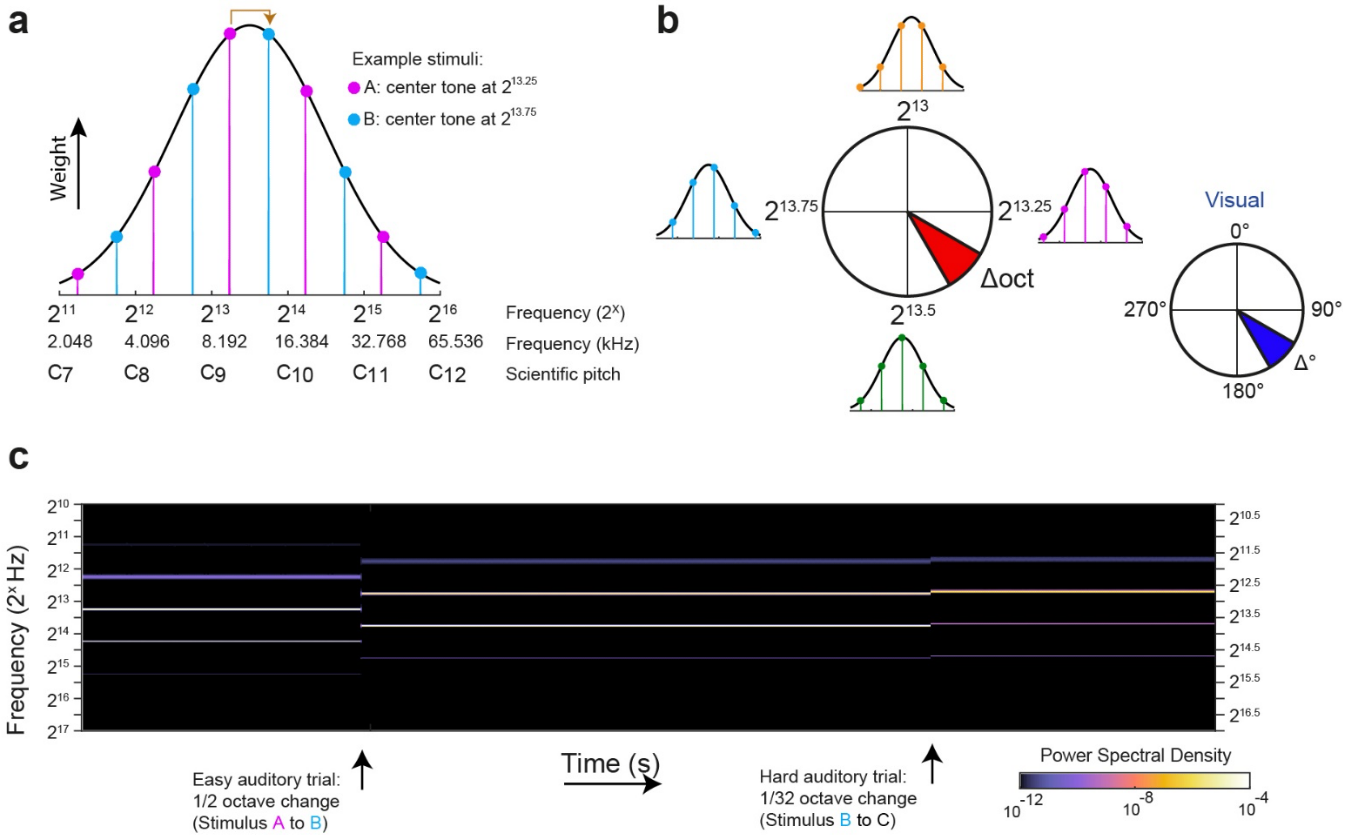

Supplementary Figure 1 Auditory stimulus design

a) Each auditory stimulus was composed of five pure tones at harmonic frequencies (octaves below and above other tones). The weight with which each tone contributed to the overall stimulus was taken from a Gaussian distribution across all possible tones. The example stimulus A in pink is composed of a tone of $2^{13.25}$ Hz (center tone, highest weight) and two lower (at $2^{11.25}$ and $2^{12.25}$ Hz) and two higher harmonics (at $2^{14.25}$ and $2^{15.25}$ Hz). Tones followed scientific pitch and are expressed as powers of two: 2^{13} corresponds to 8.192 kHz, and C_9 in scientific pitch notation. During an auditory trial, the stimulus changed to a stimulus of five new harmonic tones with different weights (for example stimulus A to B).

b) The center diagram shows the circular arrangement of all stimuli. For each cardinal direction the insets show the tonal weights associated with these stimuli. Note how ever increasing the center tone frequency ultimately results in a circular shift back to the starting stimulus. This feature is exploited in the Shepard illusion, but note that our stimuli were static and had no illusory component. This circularity can also be seen in panel *a*: going up and down

half an octave from stimulus A always results in stimulus B. The auditory stimulus set is therefore circular and mirrors the visual stimulus set with drifting gratings in all orientations (inset for comparison in right lower corner). The amount of frequency change (expressed in partial octaves, red) or orientation change (expressed in degrees, blue) determined saliency.

c) Spectrogram over time including two auditory change trials. Auditory stimuli continued to be presented until the next auditory change, which could be identified based on a difference in spectral content, and experienced as an increase or decrease in pitch. The example shows an easy auditory trial (salient change; stimulus A to B, half an octave) followed later by a difficult trial (subtle change; 1/32 of an octave). Note that this is only a schematic depiction, hence time is depicted in arbitrary units.

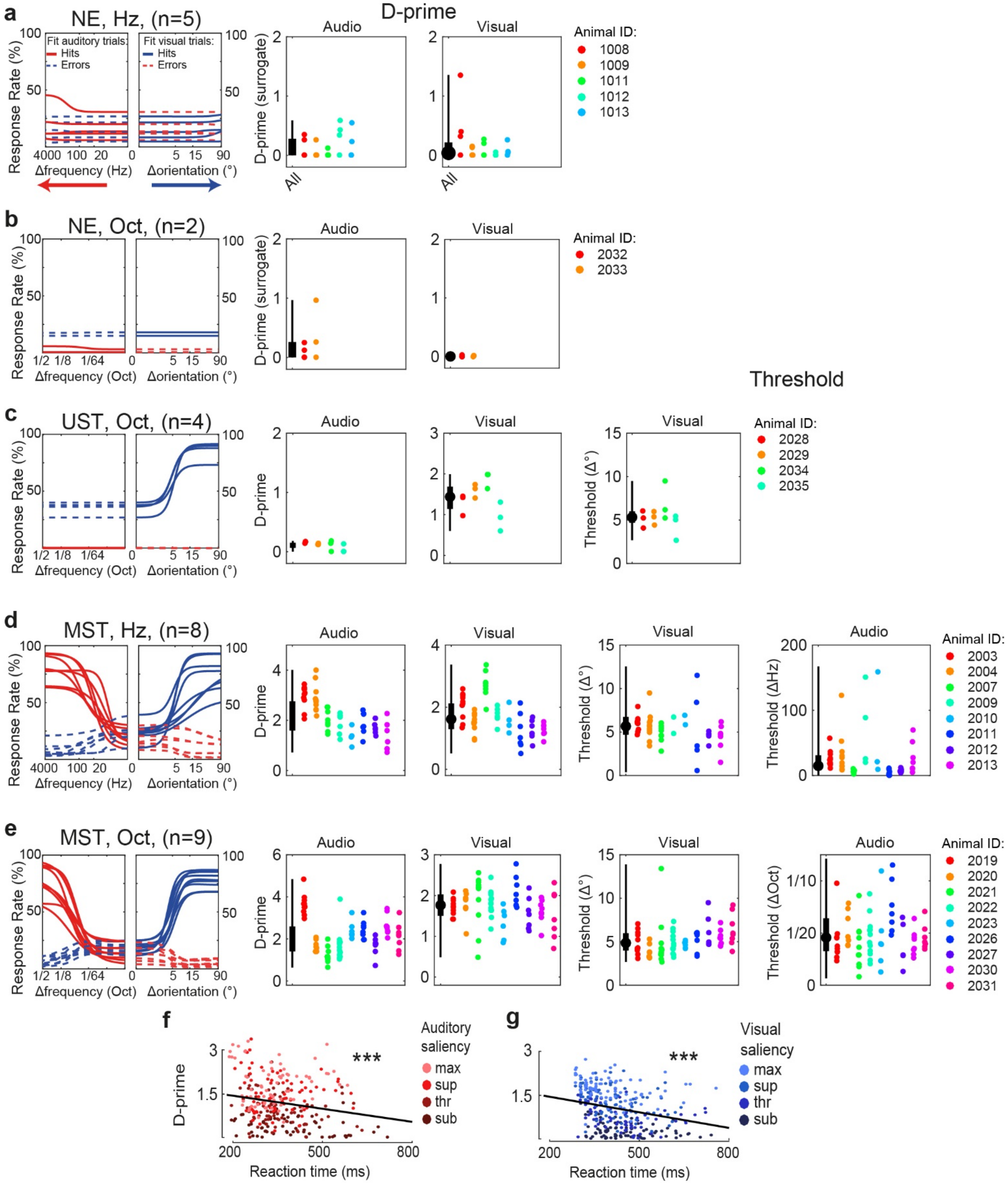

Supplementary Figure 2 Detailed psychophysical performance across versions of task A. This figure shows the data for individual animals and individual sessions for each variant of task A. We implemented two versions of auditory stimuli (frequency changes expressed in the amount of Hertz, or octaves, see Methods) and split figures

here based on version. The figure follows the same conventions as Fig. 1c-e in the main text. Dots in D-prime graphs indicate individual sessions from a single mouse (identified by color). **a** Animals (N=5) were used for the noncontingently exposed (NE) variant with auditory changes in Hz. The two leftmost panels show fitted psychometric functions (two-alternative detection model) displaying behavioral response rates at increasing levels of auditory change (left panel) and increasing levels of visual orientation change (right panel). Solid lines are hits and dotted lines are errors. Blue indicates responses to the visual lick spout and red responses to the auditory lick spout. These baseline lick rates after stimulus changes in NE mice result from the animal spontaneously licking (some licks were rewarded, but this was not temporally related to the stimuli, see Methods). Sometimes licks were emitted accidentally, briefly after a change in stimulus ('surrogate hits'). The subpanels to the right side show for each animal (different rainbow colors) and for each session (single dots) the parameters of the single session fits for the asymptotic d-prime for auditory detection (left) and visual detection (right). Within each subpanel, the boxplot shows the median (dot), interquartile range (box limits) and minima and maxima (whiskers). **b-e** Same as (**a**), but for the other reward contingencies for task A (UST and MST). For animals trained to report either visual or auditory changes, the detection thresholds are also shown. These include the visual threshold in UST and the visual and auditory threshold in MST mice. Thresholds for non-rewarded conditions were higher than the maximum saliency or infinite. No mice were trained for the UST variant of the task with auditory changes in Hz. **f** In MST animals, d-prime decreases as a function of reaction time for auditory conditions ($F(1,298) = 10.43$, $p=0.00138$; ANOVA). Each dot is one saliency condition within a single session. **g** Same as (**f**), but for the negative correlation between reaction time and d-prime in all visual conditions across combined UST and MST sessions ($F(1,268) = 11.36$, $p=0.00086$; ANOVA).

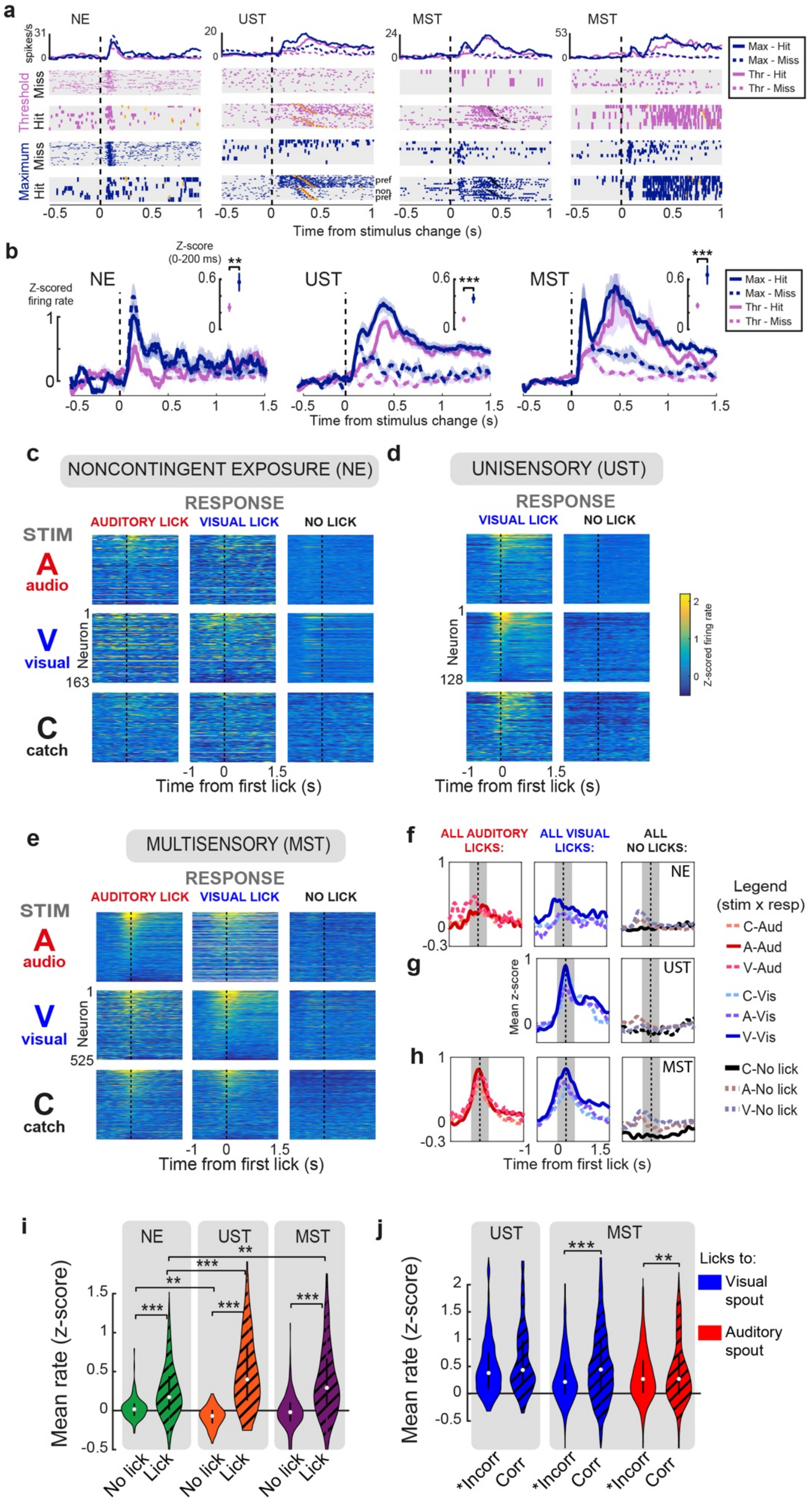

a
NE
UST
MST
MST
Max - Hit
Max - Miss
Thr - Hit
Thr - Miss
Threshold
Maximum
Miss
Hit
spikes/s
pref
non pref
Time from stimulus change (s)
b
Z-scored firing rate
Z-score (0-200 ms)
**
***
***
Time from stimulus change (s)
c
NONCONTINGENT EXPOSURE (NE)
RESPONSE
AUDITORY LICK
VISUAL LICK
NO LICK
STIM
A
audio
V
visual
C
catch
Neuron
163
Time from first lick (s)
d
UNISENSORY (UST)
128
Z-scored firing rate
e
MULTISENSORY (MST)
525
f
ALL AUDITORY LICKS:
ALL VISUAL LICKS:
ALL NO LICKS:
g
h
Mean z-score
Legend (stim x resp)
C-Aud
A-Aud
V-Aud
C-Vis
A-Vis
V-Vis
C-No lick
A-No lick
V-No lick
i
Mean rate (z-score)
No lick
Lick
j
Licks to:
Visual spout
Auditory spout
*Incorr
Corr

Supplementary Figure 3 Early and late wave dynamics across levels of change saliency and aligned to lick (Task A). a Raster plots for four example neurons showing early sensory-driven transients and late activity both for threshold and maximal orientation changes. Orange ticks indicate first lick and immediate reward delivery. Same conventions as in Fig. 2. **b** Averaging Z-scored firing rate across all neurons again reveals early sensory-driven activity in all cohorts, while hits are associated with a strong late increase in activity only in UST and MST mice for both visual saliency levels. The amplitude of early sensory-driven activity (average activity 0-200 ms, hits and misses combined) was smaller for threshold than for maximal changes for all three cohorts (shown in insets; NE: $F(1,326)=7.27$, $p=0.00739$; UST: $F(1,256)=20.43$, $p=9.47\times10^{-6}$; MST: $F(1,1568)=35.90$, $p=2.57\times10^{-9}$; Linear Mixed Model ANOVA). Lines and shading indicate mean ± SEM across neurons. **c-e** We computed the average z-scored activity across all recorded neurons in V1 aligned to the first lick for 9 stimulus-response combinations: three stimulus type conditions (A=auditory, V=visual, and C=catch – i.e. no change) and three response options (lick to visual spout, lick to auditory spout, and no lick). In NE mice, all conditions with licking (left 6 heatmaps) showed slight modulations of activity around licks, which were absent in the three conditions without licking (right 3 heatmaps). This lick-aligned modulation, however, was much more prominent in MST and UST mice (panels (**d**) and (**e**), respectively). For trials in which there was no lick, activity was aligned to the median response latency from all other trials. Conditions for which not enough trials were present to compute a reliable mean z-score for that neuron (fewer than 3 trials), were omitted from the heatmap. Therefore, trial-averaged estimates for licks to the auditory spout are absent in UST animals (trained on vision only), as animals rarely responded to the never-rewarded auditory lick spout. **f-h** All conditions with licking (left 2 panels for each row) show activity modulations hundreds of milliseconds before and after lick-onset. Each plot combines three conditions from the heatmaps in c-*e* (taken per column), and shows the Z-scored firing rate averaged over neurons (f: NE; g: UST; h: MST). Lines show mean across neurons. As in (**d**), not enough events were available to calculate licks to the auditory lick spout in UST animals. **i** Licks evoked consistently higher V1 firing activity than no-licks for all cohorts (ANOVA, n=163+128+525 neurons, $F(1,1966)=379$, $p=2.13\times10^{-7}$) in the time window around lick onset (-300 ms to +300 ms relative to lick onset, gray-shaded patch in (**f-h**). Lick modulation was stronger for trained cohorts versus naive mice (ANOVA, UST vs NE, MST vs NE, $F(2,1966)=6.08$, $p=0.002$); *$p<0.05$, **$p<0.01$, ***$p<0.001$. Violinplots show distribution of rates across neurons. Inner white dot and black line show median and interquartile range. **j** Correct licks were associated with higher V1 firing activity than incorrect licks in MST mice (ANOVA; UST, n=128, $F(1,256)=2.59$, $p=0.109$; MST n=525, visual, $F(1,1371)=140$, $p=2.54\times10^{-8}$, auditory, $F(1,1371)=9.67$, $p=0.002$).

*Incorrect licks include both false alarms and mistaken licks to opposite spout (e.g. visual stimulus, lick to auditory lick spout). Conditions are separated to allow comparing between licks to the same spout. Same style as **(i)**.

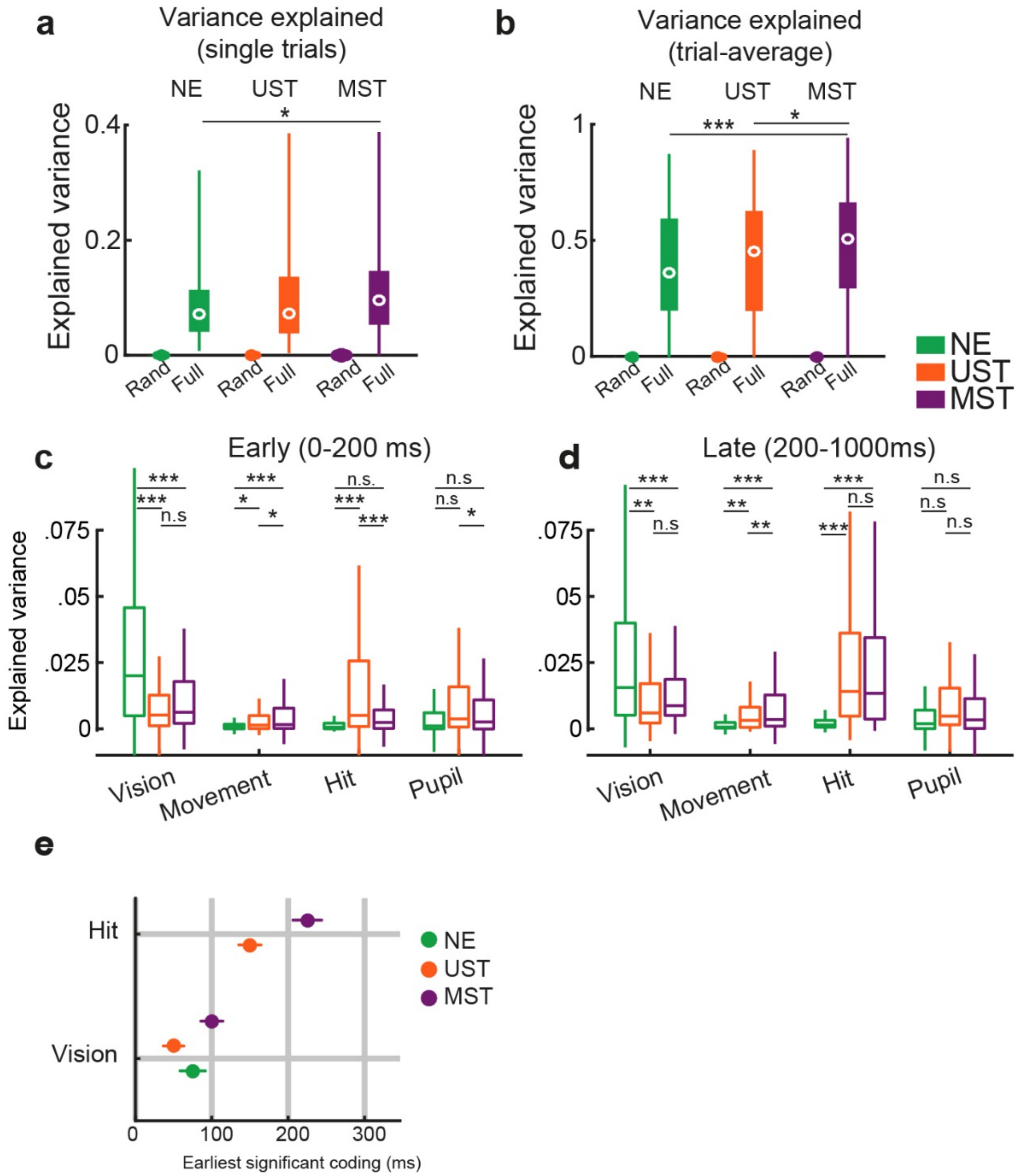

Supplementary Figure 4 Application of the generalized linear encoding model to explain variance of firing rates from different cohorts (Task A). a We constructed two models. The first model operated as a null model and consisted of a random predictor only (Rand). The second model included all predictors (Full). We quantified the model performance in two ways. First, we computed the cross-validated explained variance (EV) over all single-trial firing rates for all neurons (n=116 NE neurons, n=128 UST neurons, n=272 MST neurons). The full

model always explained significantly more variability than the random model (all $p<1e-20$), and explained variance was slightly higher for V1 neurons from MST mice than NE neurons (Linear Mixed Model ANOVA; $F(2,516) = 4.71$, $p=0.01$, ANOVA; Posthoc comparisons: MST vs NE: $F(1,513)=8.28$, $p=0.004$. NE vs UST: $F(1,513)=0.81$, $p=0.36$; MST vs UST: $F(1,513)=3.62$, $p=0.058$. Boxplots show the median (dot), interquartile range (box limits) and minima and maxima (whiskers). **b** We also quantified model performance by computing the EV of the firing rate averaged over the five stimulus x choice conditions that captured nearly all trials[20,38]. Again, the full model explained significantly more variability than the random model (all $p<1e-33$), and explained variance was higher for MST compared to NE and UST neurons (Linear Mixed Model ANOVA: $F(2,516) = 7.01$, $p=0.001$, ANOVA; Posthoc comparison: MST vs NE: $F(1,513)= 12.55$, $p<0.001$; NE vs UST: $F(1,513)=1.40$, $p=0.23$; MST vs UST: $F(1,513)=5.06$, $p=0.025$. Same style as *a*. **c** We computed the variance of the firing rate as explained by each subset of predictors for each of the task versions over time (cf. Fig. 3c). During the early post-stimulus window (averaging EV over 0-200 ms) visual predictors explained more variance in NE than UST and MST mice. (ANOVA, n=116 NE neurons, 128 UST neurons, 272 MST neurons, *$p<0.05$, **$p<0.01$, ***$p<0.001$). Boxplots show the median and interquartile range (box limits) and 1 x interquartile range (whiskers). **d** Same as (**c**), but for the late window (200-1000 ms). The hit predictor now explains more variance than during the early period and explains more variance in both UST and MST than in NE mice ($p<0.001$). Therefore, knowing when licks occurred and whether the trial was a hit or not, contributed to predicting late V1 firing in UST and MST mice. Moreover, visual predictors continue to make strong contributions in the late phase across the three training cohorts. **e** The onset latency for significant EV by visual and hit predictors. Note the difference in onset latency of hit coding between UST and MST (corresponding to the ROC analysis in Fig. 4d). Shown are mean ± SEM across neurons. (UST n=128, MST n=272 neurons, ANOVA, $F(1,337) = 1.54$, $p=0.21$).

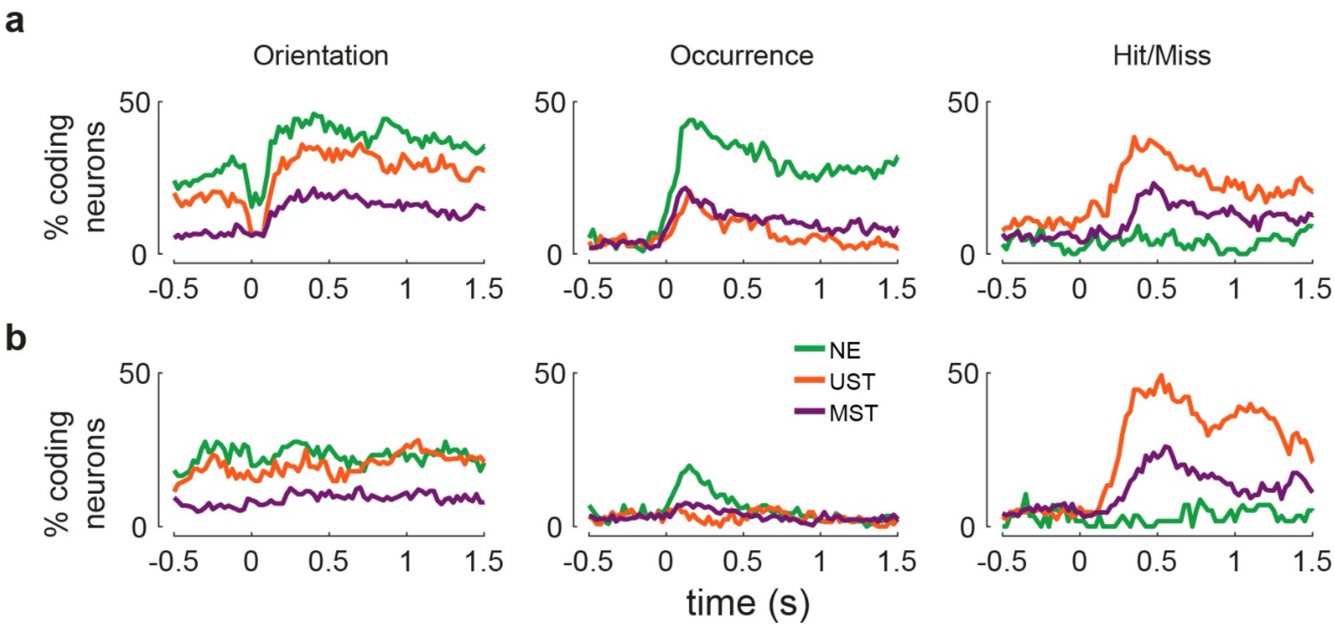

Supplementary Figure 5 Single neuron coding over time (Task A). a Percentage of V1 neurons significantly encoding selected variables over time for visual changes of maximal saliency. The strength of encoding (AUC value above shuffled) gave similar results as the fraction of coding neurons (shown here). **b** Same as (**a**), but for threshold visual changes.

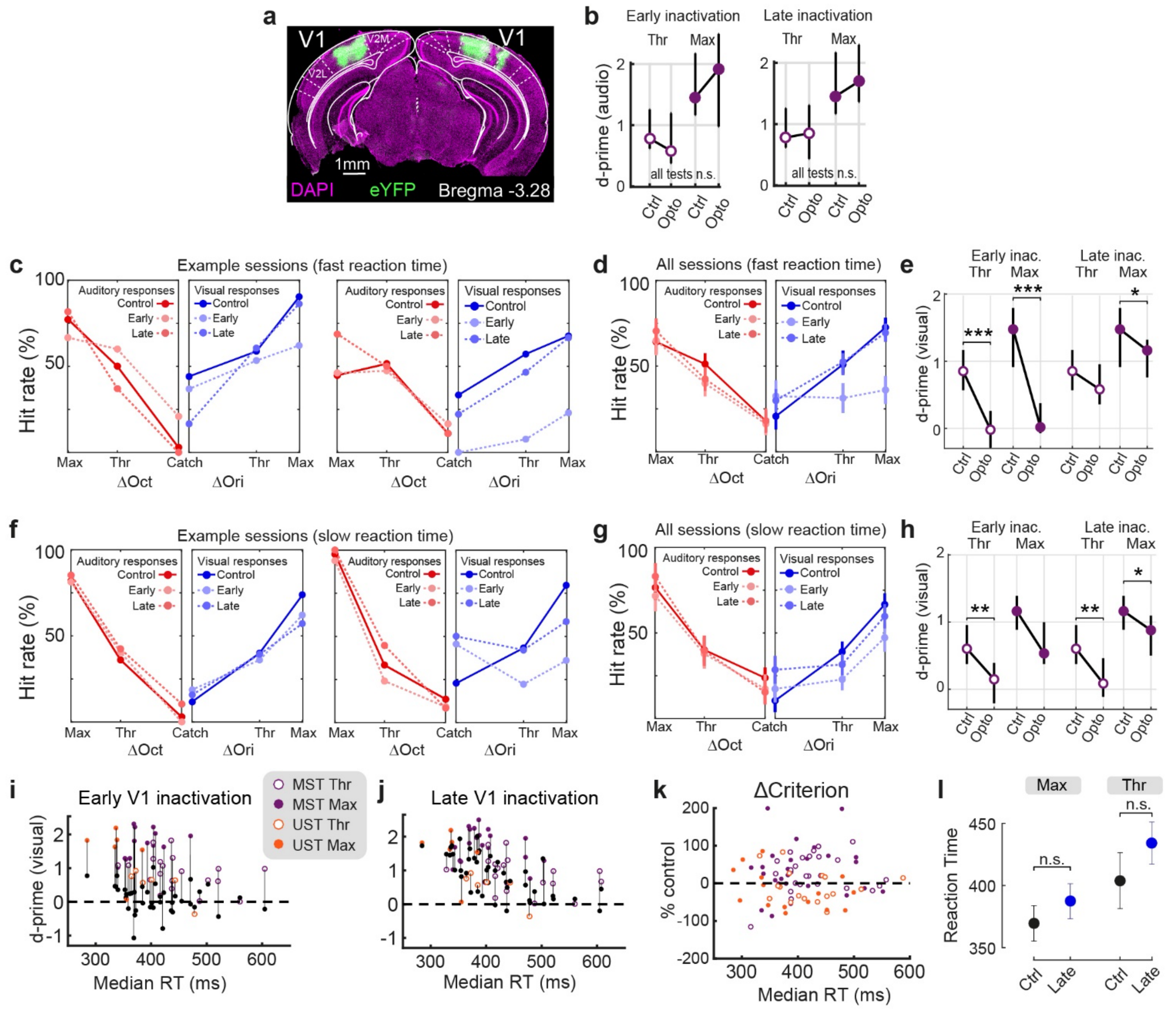

Supplementary Figure 6 Detailed characterization of early and late V1 inactivation in task A. a Coronal histological section revealing localized bilateral expression in V1. V2L: lateral secondary visual cortex. V2M: medial secondary visual cortex. Histological analyses were repeated with comparable results for all 28 mice. **b** Neither early nor late silencing affected auditory discrimination performance (d-prime) for both threshold and maximum saliencies and across UST and MST cohorts (n=34 sessions, ANOVA, all F<6, all p>0.1). **c** Behavioral detection rates for two example sessions from MST mice with fast reaction times (median visual hit reaction time 359 and 362 ms). **d** Same as Fig. 5f, but for sessions with fast reaction times (top half of all reaction times). **e** Effect of early and late inactivation on d-prime for fast sessions, as a function of visual salience (thr vs. control and max vs. control. Asterisks indicate the result of a Linear Mixed Model ANOVA: *p<0.05, **p<0.01,***p<0.001.

Exact p values: Visual thr change, Ctrl vs. Early: p=0.000027; Visual max change, Ctrl vs. Early: p=0.000002; Visual thr change, Ctrl vs. Late: p=0.606787; Visual max change, Ctrl vs. Late: p=0.022190. **f** Same as (**d**) for two example sessions from MST mice with slow reaction times (median visual hit reaction time 435 and 463 ms). **g** Same as (**d**) but for sessions with slow reaction times (bottom half of all reaction times). **h** Same as (**e**) but for sessions with slow reaction times. Exact p values: Visual thr change, Ctrl vs. Early: p=0.007136; Visual max change, Ctrl vs. Early: p=0.111827; Visual thr change, Ctrl vs. Late: p=0.001313; Visual max change, Ctrl vs. Late: p=0.0047151. Panels (**c-h**) jointly show how late silencing affects behavioral performance in slow sessions, but not fast sessions. **i** Scatter plot of visual d-prime on control (colored) and photostimulation trials (black). One data point is one session. Data points from the same session are connected with a line to visualize the reduction in d-prime. **j** Same as (**i**) but for late silencing. Note how sessions with short reaction time are proportionally less affected than sessions with long reaction time. Quantification of this effect as the percentage reduction in d-prime is in Fig. 3i-j. **k** We tested whether late inactivation could affect motivation by changing the criterion parameter in our signal detection framework (see Methods). The reduction in visual criterion by late photostimulation was not significantly correlated to the median reaction time on control trials in the same recording session ($F(1,39)=1.55$, $p=0.22$). Similarly, we found no effect when we repeated this analysis on the false alarm rate directly ($F(1,46)=0.05$, $p=0.82$). **l** As late photostimulation partially reduced hit rate for visual changes in MST mice depending on reaction time (Fig. 3h, j), some visual changes were still detected. Visual hits with and without late photostimulation were not associated with a significant difference in reaction times (MST – *max*: n=1185 trials, $F(1,1179)=2.95$, $p=0.09$; MST – *thr*: n=761 trials, $F(1,761)=0.28$, $p=0.60$; Linear Mixed Model ANOVA). Traces show mean ± SEM across visual hits. *$p < 0.05$.

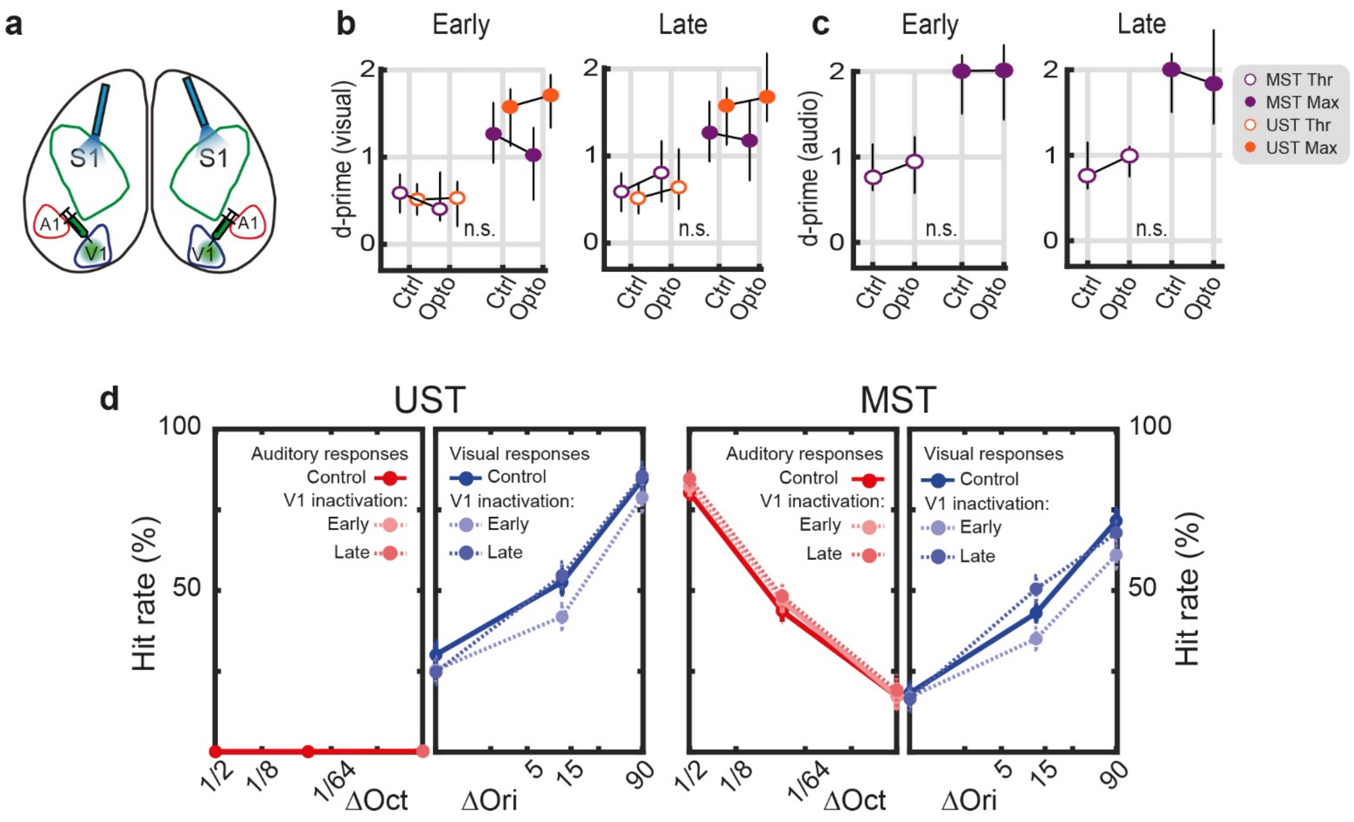

Supplementary Figure 7 Illumination of control area S1 in task A has no behavioral effects. a Control experiment with positioning of the optical fiber over uninfected S1. **b** D-prime across visual conditions. Neither early nor late S1-illumination significantly affected visual detection performance for UST or MST mice (ANOVA, n= 29 sessions, all F<6, all p>0.05, corrected for 4 multiple comparisons (Bonferroni-Holm)). **c** Same as (**b**), but for auditory conditions. Neither early nor late S1-illumination significantly affected auditory detection performance (n= 29 sessions, all p>0.05). For **b** and **c**, errorbars denote inter-quartile range. **d** Behavioral response rates for UST (left) and MST (right) mice for control, early and late S1-illumination trials.

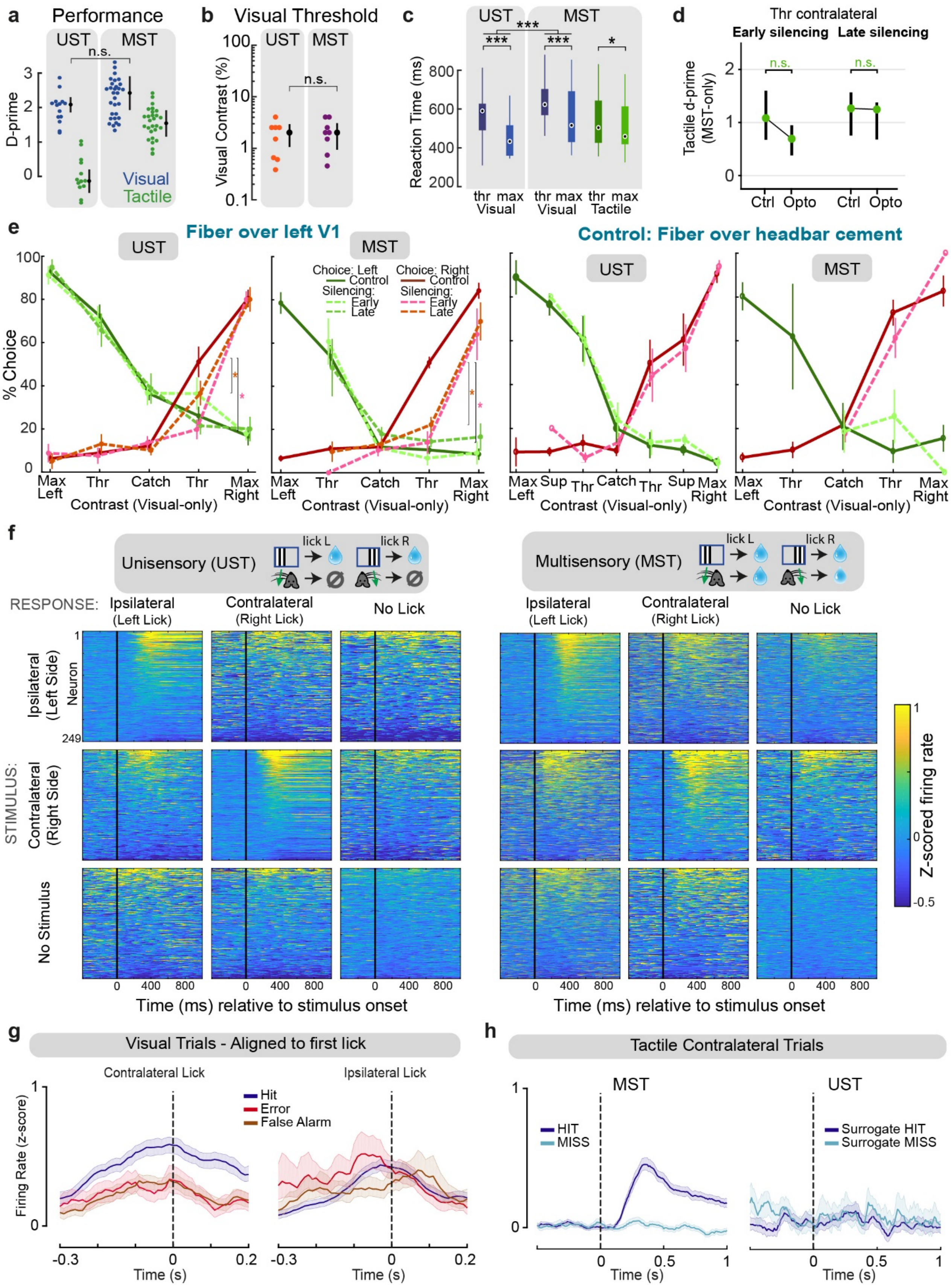

a
Performance
UST
MST
n.s.
D-prime
Visual
Tactile
b
Visual Threshold
Visual Contrast (%)
c
***
*
Reaction Time (ms)
thr max
Visual
Tactile
d
Thr contralateral
Early silencing
Late silencing
Tactile d-prime (MST-only)
Ctrl Opto
e
Fiber over left V1
Control: Fiber over headbar cement
% Choice
Choice: Left
Choice: Right
Control
Silencing:
Early
Late
Max Left
Thr
Catch
Sup
Max Right
Contrast (Visual-only)
f
Unisensory (UST)
Multisensory (MST)
lick L
lick R
RESPONSE:
Ipsilateral (Left Lick)
Contralateral (Right Lick)
No Lick
STIMULUS:
Ipsilateral (Left Side)
Contralateral (Right Side)
No Stimulus
Neuron
Z-scored firing rate
Time (ms) relative to stimulus onset
g
Visual Trials - Aligned to first lick
Contralateral Lick
Ipsilateral Lick
Hit
Error
False Alarm
Firing Rate (z-score)
Time (s)
h
Tactile Contralateral Trials
MST
UST
HIT
MISS
Surrogate HIT
Surrogate MISS

Supplementary Figure 8 Effects of optogenetic V1 silencing on visuotactile behavior (task B). a D-prime at maximum saliency for visual and tactile detection. Each dot represents one session and either right or left side detection performance. Visual performance was comparable for UST and MST mice (ANOVA, n=22 sessions, 8 mice, $F(1,44)=3.18$, $p=0.0814$). Note that null d-prime for tactile detection is expected for UST mice. For panels **a**, **b**, **d**, **e**, errorbars denote inter-quartile range. **b** Visual contrast detection thresholds were comparable for UST and MST mice (ANOVA, n=8 mice, $F(1,16)=0.3675$, $p=0.5529$). Computed for each mouse from psychometric fit, for both right and left side detections. **c** Median reaction times for each rewarded condition for threshold and maximum levels of saliency (right and left sides pooled together). Boxplots show the median and interquartile range (box limits) and 1.5 x interquartile range (whiskers). Visual reaction times were significantly shorter for UST compared to MST (ANOVA, n=1395 trials, $F(1,1391)= 54.075$, $p=3.28\times10^{-13}$, corrected for 2 multiple comparisons (Bonferroni-Holm)). *: $p<0.05$; ***: $p<0.001$). Note that tactile and visual reaction times were similar, ruling out the possibility of a sequential detection strategy where one modality would be sampled before the other one. **d** D-prime of contralateral tactile detection at threshold saliency. V1 silencing did not affect tactile performance (ANOVA, Early silencing: n=6 sessions, $F(1,12)=3.78$, $p=0.1513$; Late silencing: n=9 sessions, $F(1,14)=0.12$, $p=0.73$). Thus, for MST, silencing late V1 activity impaired visual but not tactile detection, indicating that late activity per se is not required for licking behavior. **e** Left: Psychometric curves for experiments with left hemisphere V1 silencing (same experiments as Fig. 6c), for visual-only trials. Data points for MST Max left are missing because these were not assessed in the experimental protocol (same for Max left and Thr left conditions in control experiments). Since monocular stimuli were used and left hemisphere V1 was silenced, potential effects were expected for stimuli on the right side (contralateral) but not on the left side (ipsilateral). (ANOVA, n=7 sessions, UST Early, $F(1,10.7)=39$, $p=0.0002$; UST Late, $F(1,10.7)$, $p=0.0178$; MST Early, $F(1,12)=32$, $p=0.0002$; MST Late, $F(1,18)=38$, $p=3\times10^{-5}$). *: $p<0.05$. Note that late silencing for UST mice had a significant effect on the percentage of right choices, but no effect on the corresponding d-prime. Right: psychometric curves for control experiments where the optic fiber was placed above the mouse headbar cement and therefore not above V1 (all not significant). Errorbars denote interquartile range. **f** Average z-scored activity for all recorded left-hemisphere V1 neurons for each of all 9 possible visual-only stimulus-response combinations for UST (left) and MST (right) trained mice. Neurons for which no more than three trials were present in the given condition were omitted. For each condition, neurons were sorted according to their mean z-score between 50 and 500 ms. **g** Average z-scored firing rate of responsive V1 neurons during visual-only trials, for three different conditions eliciting the

same licking response (left: contralateral lick, right: ipsilateral lick), showing that late V1 activity cannot be explained by licking alone (see also Supplementary Fig. 3). Activity was aligned to the first lick in the response window. Licks made to the wrong side were termed “errors”. Same neurons as in Fig. 6c. Shaded area: bootstrapped 95% confidence intervals. **h** Average z-scored firing rate of responsive V1 neurons during contralateral tactile trials, split by choice, for UST and MST mice. For UST, surrogate hits correspond to licks to the same side of the tactile stimulus (although unrewarded) and surrogate misses correspond to trials without licks. Late activity was present only in MST Hits, indicating that the same stimulus and the same behavioral response triggered late activity in a context-dependent manner. Shaded area: bootstrapped 95% confidence intervals.

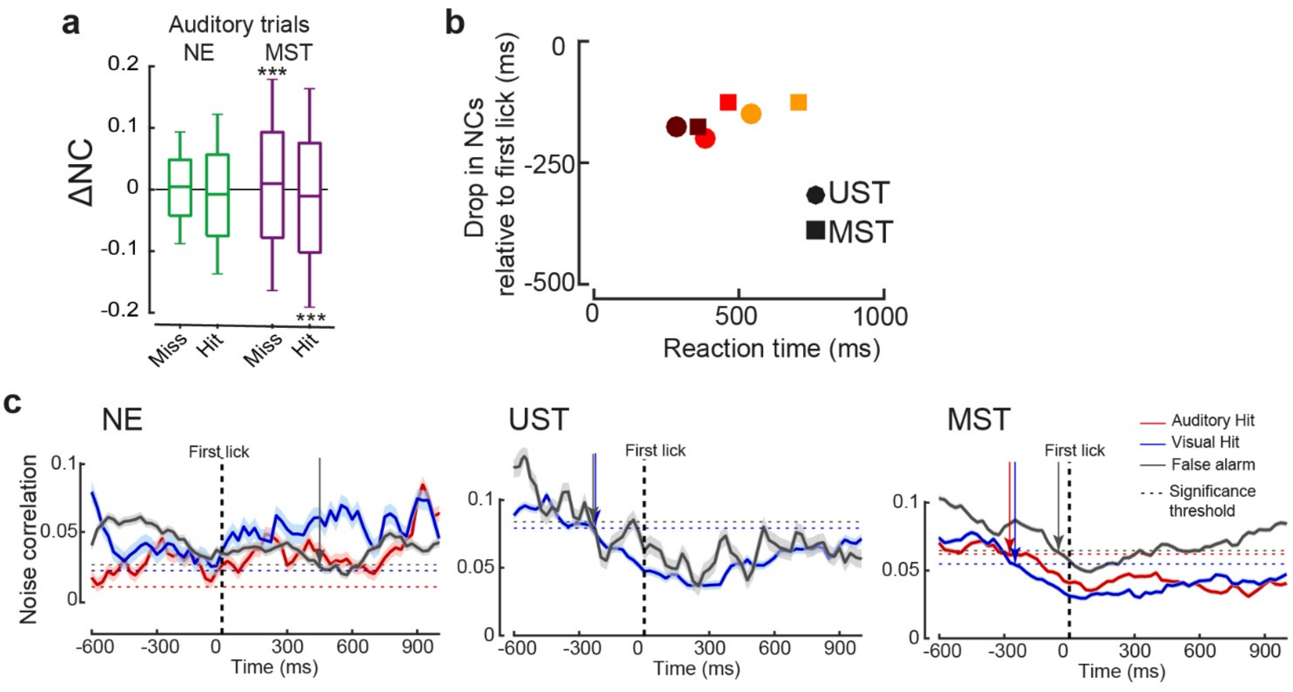

Supplementary Figure 9 Noise correlations in auditory trials and latency of the drop in noise correlations during visual trials in task A. a Decrease in noise correlation (NC) of V1 cell pairs with respect to baseline for auditory hits and misses across cohorts (same conventions as Fig. 5c). For UST mice there were too few auditory hits to compute noise correlations. In MST mice, noise correlations decreased during hits (ANOVA, n=14462 trials, $F(1,28847) = 99.90$, $p = 1.7*10^{-23}$) and increased during misses (ANOVA, n=13656 trials, $F(1,27395) = 22.61$, $p=1.99*10^{-6}$). The fact that NCs also decrease in auditory hits indicates that the drop in NCs in the visual cortex is not specific to visual trials, and possibly is a more general mechanism that may subserve decision making. Boxplots show the median and interquartile range (box limits) and 1 x interquartile range (whiskers). **b** Drop in NCs during visual trials, but relative to the first lick for each tertile of reaction times for UST and MST. Same as Fig. 5f, but aligned to reaction time. No significant correlation is found (p=0.09), in contrast with Fig. 5f, indicating that the drop in NCs precedes reaction time by a relatively constant time lag. **c** Noise correlations (NC) of V1 cell pairs over time aligned to the first lick for the different trial types and cohorts. Dotted line shows the threshold for a significant drop in NCs with respect to baseline (-1000 to -500 ms relative to first lick). Noise correlations decreased most for visual hits in visually trained mice (UST and MST), but not in NE mice. For UST mice there were too few auditory hits to compute noise correlations. Shaded area corresponds to s.e.m.
