# Supplementary material for: Multisensory task demands temporally extend the causal requirement for visual cortex in perception
Source: Nat Commun. 2022 May 23;13:2864. doi: 10.1038/s41467-022-30600-4 (PMC9126973; doi:10.1038/s41467-022-30600-4)
Supplement: Supplementary file 2 — Reporting Summary [file 41467_2022_30600_MOESM2_ESM.pdf]

nature portfolio

Corresponding author(s): Umberto Olcese

Last updated by author(s): Apr 13, 2022

Reporting Summary

Nature Portfolio wishes to improve the reproducibility of the work that we publish. This form provides structure for consistency and transparency in reporting. For further information on Nature Portfolio policies, see our Editorial Policies and the Editorial Policy Checklist.

Statistics

For all statistical analyses, confirm that the following items are present in the figure legend, table legend, main text, or Methods section.

n/a	Confirmed	
☐	☒	The exact sample size (*n*) for each experimental group/condition, given as a discrete number and unit of measurement
☐	☒	A statement on whether measurements were taken from distinct samples or whether the same sample was measured repeatedly
☐	☒	The statistical test(s) used AND whether they are one- or two-sided<br>*Only common tests should be described solely by name; describe more complex techniques in the Methods section.*
☐	☒	A description of all covariates tested
☐	☒	A description of any assumptions or corrections, such as tests of normality and adjustment for multiple comparisons
☐	☒	A full description of the statistical parameters including central tendency (e.g. means) or other basic estimates (e.g. regression coefficient) AND variation (e.g. standard deviation) or associated estimates of uncertainty (e.g. confidence intervals)
☐	☒	For null hypothesis testing, the test statistic (e.g. *F*, *t*, *r*) with confidence intervals, effect sizes, degrees of freedom and *P* value noted<br>*Give P values as exact values whenever suitable.*
☒	☐	For Bayesian analysis, information on the choice of priors and Markov chain Monte Carlo settings
☐	☒	For hierarchical and complex designs, identification of the appropriate level for tests and full reporting of outcomes
☒	☐	Estimates of effect sizes (e.g. Cohen's *d*, Pearson's *r*), indicating how they were calculated

Our web collection on statistics for biologists contains articles on many of the points above.

Software and code

Policy information about availability of computer code

Data collection	-custom GNU Octave 4.x code (behavioral data acquisition)<br>-custom Arduino code compiled in Arduino IDE 1.0.8 and running on an Arduino Uno board (behavioral data acquisition)<br>-Cheetah 5.0 in combitiona with Digital Lynx SX Acquisition system by Neuralynx (neural data acquisition)
Data analysis	-Klusta 3.0.16, open source software for automatic spike sorting (https://klusta.readthedocs.io/en/latest/) (neural data preprocessing)<br>-Phy 1.0.9, open source software for manual curation of spike sorted data (https://phy.readthedocs.io/en/latest/) (neural data preprocessing)<br>-DeepLabCut 2.1.10, for animal pose estimation of video data (https://github.com/DeepLabCut/DeepLabCut) (behavioral data analysis)<br>-custom written MATLAB code (r2016a, Mathworks) (behavioral and neural data analysis)<br>-custom written Python code (behavioral and neural data analysis)<br>-Scikit-learn Python toolbox, version 0.23.0 (neural data analysis)<br>-Glmnet package in Matlab (2015 version)<br>-All the custom Matlab and Python code that we developed to analyze the data and prepare the figures in the manuscript is openly available at https://gitlab.com/csnlab/olcese-lab/modid-project/2nd-bump.

For manuscripts utilizing custom algorithms or software that are central to the research but not yet described in published literature, software must be made available to editors and reviewers. We strongly encourage code deposition in a community repository (e.g. GitHub). See the Nature Portfolio guidelines for submitting code & software for further information.

Data

Policy information about availability of data

All manuscripts must include a data availability statement. This statement should provide the following information, where applicable:

- Accession codes, unique identifiers, or web links for publicly available datasets
- A description of any restrictions on data availability
- For clinical datasets or third party data, please ensure that the statement adheres to our policy

The behavioral and neural data supporting the findings of this study is openly available at https://gitlab.com/csnlab/olcese-lab/modid-project/2nd-bump.

Field-specific reporting

Please select the one below that is the best fit for your research. If you are not sure, read the appropriate sections before making your selection.

☒ Life sciences ☐ Behavioural & social sciences ☐ Ecological, evolutionary & environmental sciences

For a reference copy of the document with all sections, see nature.com/documents/nr-reporting-summary-flat.pdf

Life sciences study design

All studies must disclose on these points even when the disclosure is negative.

Sample size	We did not predetermine the sample size. For each experimental group we collected data from at least 4 mice, as this minimal sample size is comparable to that used studies in the field. For example of recent publications using approaches similar to ours, see:<br>- Resulaj, Arbora, et al. "First spikes in visual cortex enable perceptual discrimination." Elife 7 (2018): e34044: N=8-12 for the different experimental groups.<br>- Zatka-Haas, Peter, et al. "Sensory coding and the causal impact of mouse cortex in a visual decision." Elife 10 (2021): e63163: N=5-7 for the different experimental groups.<br>- Kirchberger, Lisa, et al. "The essential role of recurrent processing for figure-ground perception in mice." Science advances 7.27 (2021): eabe1833: N=3-13 for the different experimental groups.<br>We did not set any maximal sample size, however we ran some analyses to verify, via a resampling approach - that our results were not a product of different sample sizes between experimental groups.
Data exclusions	-Some subjects were unable to successfully learn the complex version of the behavioral task and to make decisions based on both modalities (MST task versions) within 2 months and were excluded from further experiments.<br>-Experimental sessions were excluded with very poor behavioral performance. Single sessions in which our behavioral model fit of the visual threshold was below 1 degree or above 45 degrees were excluded (average threshold ±6 degrees, n=3/179 sessions excluded). This exclusion criteria was defined posthoc to prevent drawing conclusions relating neural signals to behavioral data performance in poor performing sessions.
Replication	We replicated our results in a second behavioral paradigm with an additional n=12 mice.
Randomization	Littermates were always assigned to the same experimental group (NE, UST, or MST), due to differences in the duration of the behavioral training phase. Photostimulation trials to inactivate primary visual cortex were randomly interleaved with control trials.
Blinding	Researchers were not blinded to the experimental group as they could not perform the experiments without knowing the condition (i.e. they needed to run a different task protocol for unisensory trained mice versus multisensory trained mice).

Reporting for specific materials, systems and methods

We require information from authors about some types of materials, experimental systems and methods used in many studies. Here, indicate whether each material, system or method listed is relevant to your study. If you are not sure if a list item applies to your research, read the appropriate section before selecting a response.

Materials & experimental systems

n/a	Involved in the study	
☒	☐	Antibodies
☒	☐	Eukaryotic cell lines
☒	☐	Palaeontology and archaeology
☐	☒	Animals and other organisms
☒	☐	Human research participants
☒	☐	Clinical data
☒	☐	Dual use research of concern

Methods

n/a	Involved in the study	
☒	☐	ChIP-seq
☒	☐	Flow cytometry
☒	☐	MRI-based neuroimaging

Animals and other organisms

Policy information about studies involving animals; ARRIVE guidelines recommended for reporting animal research

Laboratory animals	Mus musculus, C57BL/6J wild-type, male, 8-40 weeks of age.<br>Mus musculus, PVcre line (B6;129P2-Pvalbtm1(cre)Arbr/J, RRID: IMSR_JAX:008069), male, 8-40 weeks of age.<br>Mice were group-housed in a pathogen-free facility under a reversed day-night schedule (lights were switched off at 8:00 and back on at 20:00). All experimental procedures were performed during the dark period. Temperature in the housing facility was maintained between 19.5 and 23.5 C, and humidity was kept in a range between 45 and 65%. Mice were subjected to a water restriction schedule and minimum weight was kept above 85% of their average weight between P60-P90. They typically earned their daily ration of liquid by performing the behavioral task but received a supplement when the earned amount was below a minimum of 0.025 ml/g body weight per 24h. Mice received ad libitum food.
Wild animals	No wild animals were used in this study
Field-collected samples	No field-collected samples were used in this study
Ethics oversight	All animal experiments were performed according to national and institutional regulations. The experimental protocol was approved by the Dutch Commission for Animal Experiments and by the Animal Welfare Body of the University of Amsterdam.

Note that full information on the approval of the study protocol must also be provided in the manuscript.
